# IFN-**α** with dasatinib broadens the immune repertoire in patients with chronic-phase chronic myeloid leukemia

**DOI:** 10.1172/JCI152585

**Published:** 2022-09-01

**Authors:** Jani Huuhtanen, Mette Ilander, Bhagwan Yadav, Olli M.J. Dufva, Hanna Lähteenmäki, Tiina Kasanen, Jay Klievink, Ulla Olsson-Strömberg, Jesper Stentoft, Johan Richter, Perttu Koskenvesa, Martin Höglund, Stina Söderlund, Arta Dreimane, Kimmo Porkka, Tobias Gedde-Dahl, Björn T. Gjertsen, Leif Stenke, Kristina Myhr-Eriksson, Berit Markevärn, Anna Lübking, Andreja Dimitrijevic, Lene Udby, Ole Weis Bjerrum, Henrik Hjorth-Hansen, Satu Mustjoki

**Affiliations:** 1 Translational Immunology Research Program and Department of Clinical Chemistry and Hematology, University of Helsinki, Helsinki, Finland.; 2 Hematology Research Unit Helsinki and Helsinki University Hospital Comprehensive Cancer Center, Department of Hematology, Helsinki, Finland.; 3 Department of Computer Science, Aalto University, Espoo, Finland.; 4 Department of Medical Sciences, Uppsala University and Hematology Section, Uppsala University Hospital, Uppsala, Sweden.; 5 Department of Hematology, Aarhus University Hospital, Aarhus, Denmark.; 6 Department of Hematology, Oncology and Radiation Physics, Skåne University Hospital, Lund, Sweden.; 7 Department of Medical and Health Sciences, Linköping University, Department of Hematology, County Council of Östergötland, Linköping, Sweden.; 8 iCAN Digital Precision Cancer Medicine Flagship, Helsinki, Finland.; 9 Department of Hematology, Oslo University Hospital, Rikshospitalet, Oslo, Norway.; 10 Department of Internal Medicine, Hematology Section, Haukeland University Hospital and Department of Clinical Science, University of Bergen, Bergen, Norway.; 11 Department of Hematology, Karolinska University Hospital and Karolinska Institutet, Stockholm, Sweden.; 12 Department of Internal Medicine, Sunderby Hospital, Luleå, Sweden.; 13 Department of Hematology, Umeå University Hospital, Umeå, Sweden.; 14 Department of Hematology, Odense University Hospital, Odense, Denmark.; 15 Department of Hematology, Zealand University Hospital, Roskilde, Denmark.; 16 Department of Hematology, Rigshospitalet, University Hospital of Copenhagen, Copenhagen, Denmark.; 17 Department of Hematology, St. Olavs Hospital, Trondheim, Norway.; 18 Department of Cancer Research and Molecular Medicine, Norwegian University of Science and Technology (NTNU), Trondheim, Norway.

**Keywords:** Hematology, Immunology, Bioinformatics, Cancer immunotherapy, Leukemias

## Abstract

In chronic myeloid leukemia (CML), combination therapies with tyrosine kinase inhibitors (TKIs) aim to improve the achievement of deep molecular remission that would allow therapy discontinuation. IFN-α is one promising candidate, as it has long-lasting effects on both malignant and immune cells. In connection with a multicenter clinical trial combining dasatinib with IFN-α in 40 patients with chronic-phase CML (NordCML007, NCT01725204), we performed immune monitoring with single-cell RNA and T cell receptor (TCR) sequencing (*n* = 4, 12 samples), bulk TCRβ sequencing (*n* = 13, 26 samples), flow cytometry (*n* = 40, 106 samples), cytokine analyses (*n* = 17, 80 samples), and ex vivo functional studies (*n* = 39, 80 samples). Dasatinib drove the immune repertoire toward terminally differentiated NK and CD8^+^ T cells with dampened functional capabilities. Patients with dasatinib-associated pleural effusions had increased numbers of CD8^+^ recently activated effector memory T (Temra) cells. In vitro, dasatinib prevented CD3-induced cell death by blocking TCR signaling. The addition of IFN-α reversed the terminally differentiated phenotypes and increased the number of costimulatory intercellular interactions and the number of unique putative epitope-specific TCR clusters. In vitro IFN-α had costimulatory effects on TCR signaling. Our work supports the combination of IFN-α with TKI therapy, as IFN-α broadens the immune repertoire and restores immunological function.

## Graphical abstract

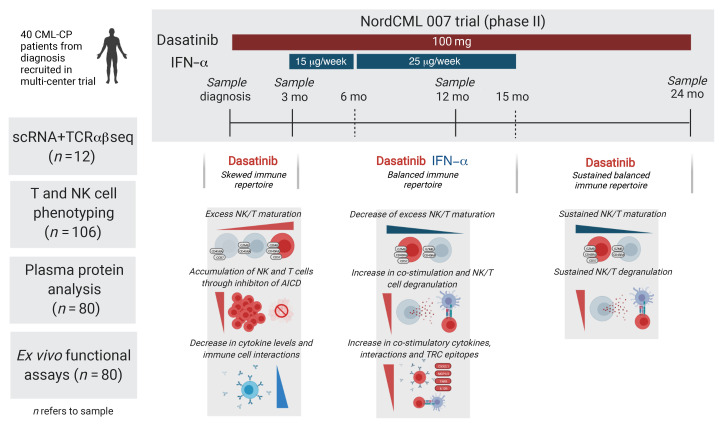

## Introduction

Currently, the ultimate therapeutic goal in patients with chronic-phase chronic myeloid leukemia (CP-CML) is to achieve deep molecular remission, which would allow the discontinuation of tyrosine kinase inhibitor (TKI) therapy and treatment-free remission in approximately half of the patients. Although treatment responses to first-generation TKI imatinib are paradigm-shifting, not all patients with CP-CML gain an optimal deep molecular response with imatinib (1). Thus, current clinical trials are aiming to improve the response rates with other TKIs and combination therapies.

Second-generation TKIs, such as dasatinib and nilotinib, are more potent BCR-ABL1 inhibitors, inducing higher response rates than imatinib (2, 3). In addition to BCR-ABL1, dasatinib inhibits other kinases, such as LCK and those in the SRC family that mediate important immunological functions. Dasatinib has shown contradictory results in in vitro and in vivo studies; in in vitro studies, it shows inhibitory effects on T and NK cells, but positive immunomodulatory effects are observed in vivo in a proportion of patients (4). We and others have demonstrated that dasatinib treatment increases the frequency of clonally expanded cytotoxic CD4^+^ and CD8^+^ T cells that are active in IFN-γ secretion and induces rapid mobilization of lymphocytes (5–7). However, due to its broad kinase inhibition profile, dasatinib can also induce immune-related adverse events, such as dermatitis and pleural effusions (PEs), which can lead to a treatment switch (2).

Before TKIs, the only regimen that could reestablish normal hematopoiesis in patients with CML was IFN-α. The combination of imatinib with IFN-α has been demonstrated to induce higher rates of molecular responses than imatinib alone in randomized controlled trials, which are ongoing with second-generation TKIs (8–10). A potential driver of this success might be the long-term immunomodulatory effects of IFN-α, which could contribute to the control or eradication of the TKI-insensitive, quiescent leukemic CML stem cells (11, 12). Supporting this, patients with increased frequencies of NK cells and more active effector memory T (Tem) cells have had higher probabilities of discontinuing IFN-α monotherapy without subsequent disease relapse (13–16), but the immunomodulatory effects of IFN-α in combination with TKIs are not well understood.

**Figure 1:**
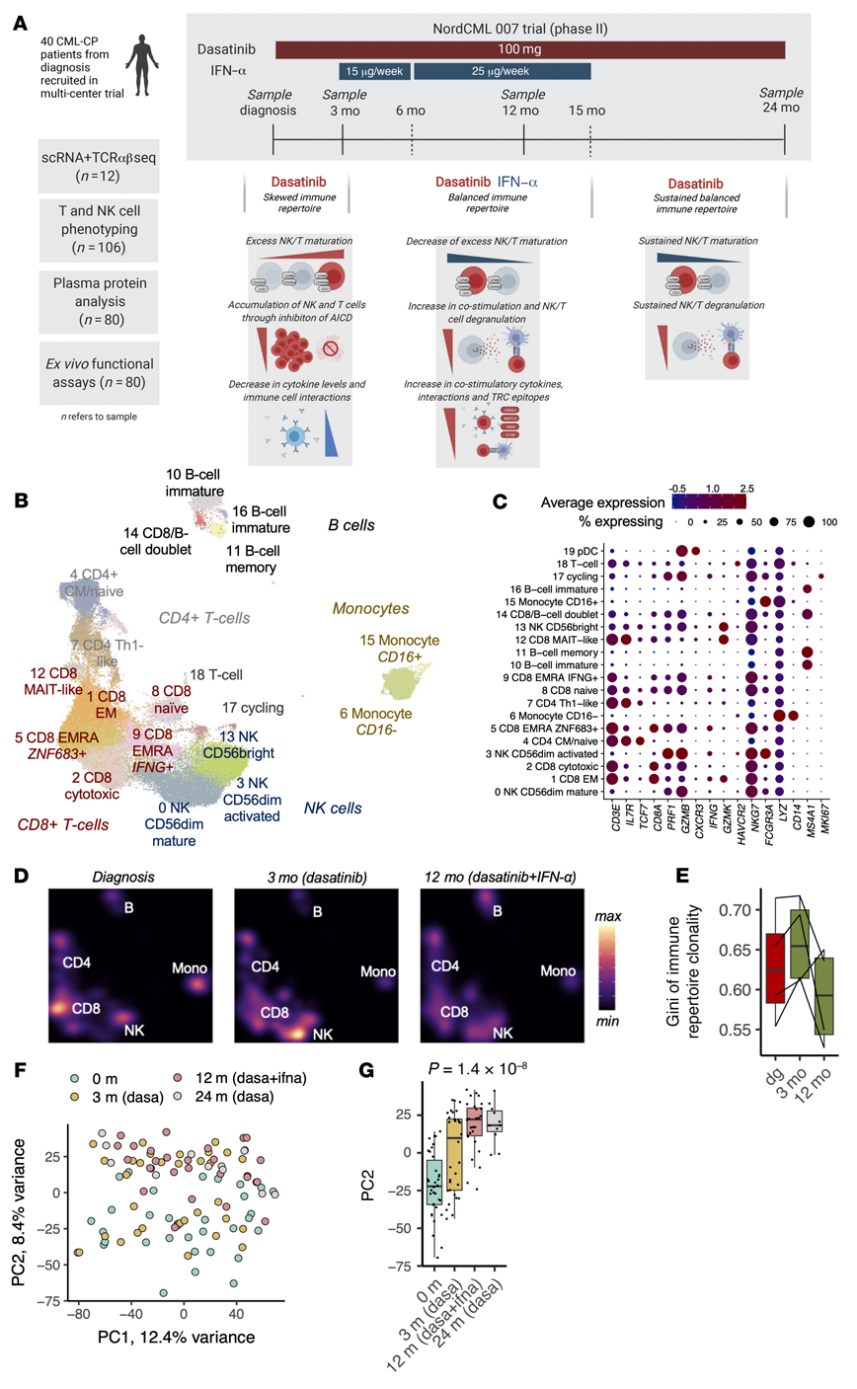
The landscape of CP-CML patients’ immune repertoire during dasatinib plus IFN-α combination treatment is dominated by NK and CD8^+^ T cells. (**A**) Schematics showing the outline of the study. Samples were taken at 0-, 3-, 12-, and 24-month time points. Figure was created with BioRender.com. (**B**) UMAP projection showing the scRNA-seq profiles from all 100,000 cells (*n* = 4, 3 time points) in the study, colored by inferred cluster. (**C**) Dot plot showing the expression of selected canonical markers aiding in cluster annotation. The color of the dot corresponds to the average expression (*z* score) of the gene and the size of the dot corresponds to the percentages of cells in the cluster expressing the gene. (**D**) The same UMAP projection as in **B** showing the cell densities at different time points. (**E**) The immune repertoire evenness at different time points calculated from scRNA-seq clusters with Gini index (higher values correspond to more skewed repertoires). (**F**) PCA plot from the flow cytometry–profiled samples (*n* = 40 patients, 4 time points). (**G**) Box-and-whisker plot (defined in the Methods) showing the position of samples in PC2. *P* value was calculated with the Kruskal-Wallis test.

Recently, 2 phase II clinical trials (NordCML007 [NCT01725204]; Dasa-PegIFN trial, [NCT01872442]) have evaluated the safety and efficacy of the combination of dasatinib with low-dose IFN-α treatment as the first line therapy in patients with CP-CML (17, 18). In both trials, the addition of IFN-α to dasatinib showed higher response rates and decreased rates of PEs in comparison with historical cohorts treated with dasatinib alone. To understand the effect of dasatinib and IFN-α combination treatment on the immune system and how it correlates with clinical parameters, we conducted a substudy of the NordCML007 clinical trial with single-cell RNA and T cell receptor (TCR) sequencing (scRNA-seq and scTCRαβ-seq), flow cytometry, bulk TCRβ-seq, plasma cytokine profiling, and ex vivo functional studies (Figure 1A). Our results show the opposing effects of dasatinib and IFN-α on the immune system and how these immunological changes can be linked to both treatment outcomes and adverse events.

## Results

### The landscape of CP-CML patients’ immune repertoire during dasatinib plus IFN-α combination treatment is dominated by NK and CD8^+^ T cells.

Overall, we recruited 40 newly diagnosed patients from 15 hospitals with CP-CML to receive 100 mg dasatinib q.d., and after 3 months of dasatinib monotherapy, IFN-α treatment was added (first 3 months 15 μg/week, then 25 μg/week of pegylated IFN-α). After 12 months of combination treatment, patients resumed dasatinib monotherapy. More detailed clinical results can be found in Supplemental Table 1 (supplemental material available online with this article; https://doi.org/10.1172/JCI152585DS1) and in the report of clinical efficacy data (17). In this immunological substudy involving patients in the clinical trial, peripheral blood (PB) samples were collected at diagnosis and 3, 12, and 24 months after the start of therapy.

To understand the immune landscape of CP-CML during dasatinib plus IFN-α treatment, we analyzed over 100,000 flow cytometry–sorted CD45^+^ blood mononuclear cells from 12 samples with scRNA-seq and scTCRαβ-seq (10× Genomics, *n* = 4; samples at 0, 3, and 12 months; 2 patients with PE and 2 patients without adverse effects; patient details in Supplemental Table 1). By utilizing deep generative modeling (19), we identified 20 clusters, all of which were shared among individuals and 6 of which were identified as CD8^+^ T cells, 4 as B cells, 3 as NK cells, 2 as CD4^+^ T cells, and 1 as monocytes (Figure 1, B and C, cluster annotation in Methods and Supplemental Figure 1, A–G, differentially expressed genes [DEGs] in Supplemental Table 2).

At diagnosis, the immune repertoire was skewed toward different CD8^+^ T cells (CD8^+^ recently activated effector memory T [Temra] cluster 5 and cytotoxic CD8^+^ effector T [Teff] cluster 2), while after 3 months of dasatinib therapy, the landscape was dominated by NK cells (mature CD56^dim^ NK cluster 3) (Figure 1D and Supplemental Figure 2, A–C). However, after the addition of IFN-α to dasatinib, the immune repertoire transformed to a more balanced distribution of different immune cell types (Figure 1D). This was noted as a higher immune repertoire richness and measured as a lower Gini index (*P* = 0.08, one-sided paired *t* test, Figure 1E) compared with a higher immune repertoire clonality after dasatinib monotherapy (*P* = 0.04). Similarly, unsupervised principal component analysis (PCA) of the flow cytometry data (*n* = 40 patients, in total 106 samples) revealed unique signatures during each treatment step (Figure 1F). In particular, PC2, which explained the second highest variation in the data (8.4%), separated the samples in a stepwise manner by time points (*P* < 0.001, Kruskal-Wallis, Figure 1G). Twelve-month (dasatinib + IFN-α combination therapy) and 24-month (dasatinib monotherapy after discontinuation of IFN-α at 15 months) samples clustered closer together than diagnosis and 3-month samples, suggesting that the addition of IFN-α causes long-lasting effects on the immune system, which can be seen even after 9 months of absence of IFN-α.

### Dasatinib treatment induces NK and CD8^+^ T cell maturation.

**Figure 2:**
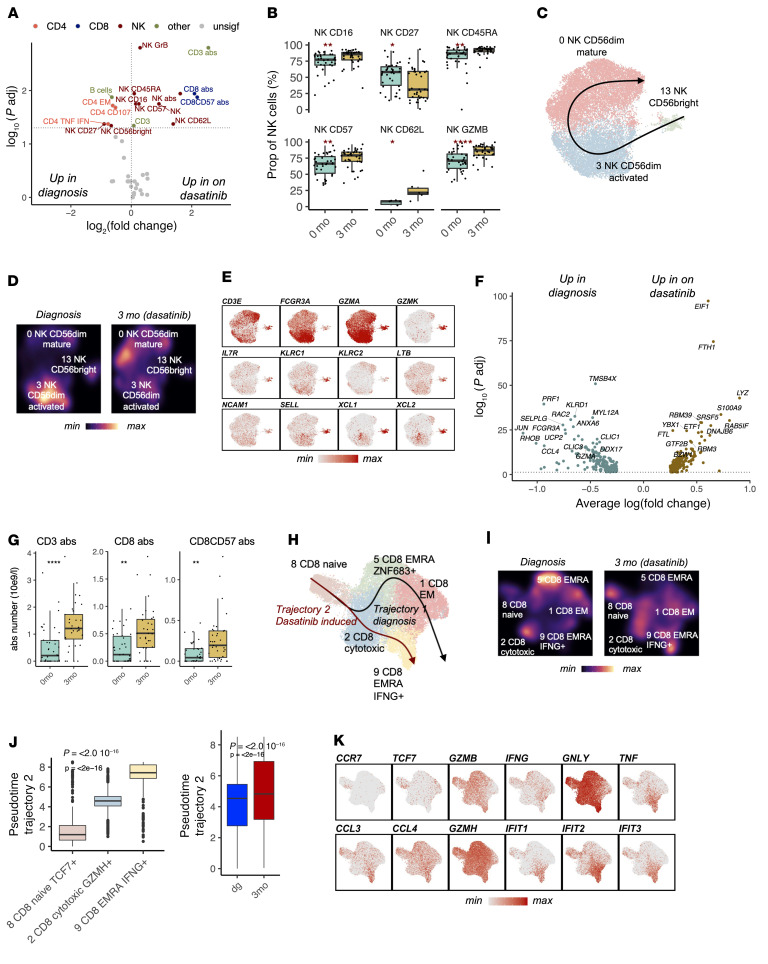
Dasatinib treatment induces NK and CD8^+^ T cell maturation. (**A**) The differentially abundant (*P_adj_* < 0.05, Benjamini-Hochberg–corrected Mann-Whitney) flow cytometry cell populations between 3 months after dasatinib and diagnosis (*n* = 40). The *x* axis denotes the log_2_-transformed fold change (log_2_FC) of median population abundances. Populations with 2 markers denote the proportion of positive cells from the host population (CD8CD57 = CD57^+^ cells from CD8^+^ cells); a single marker (e.g., T cells) denotes the proportion of these cells from lymphocytes; “abs” denotes absolute cell numbers. (**B**) Selected NK cell subpopulations as percentages of total NK cells analyzed with flow cytometry (*n* = 40). The *P* values were calculated with the Mann-Whitney test. (**C**) UMAP projection of the NK cell clusters identified with scRNA-seq (*n* = 4) in Figure 1B. The superimposed line represents the predicted maturation trajectory. (**D**) The same as in **C** showing the cell densities at different time points. (**E**) The same as in **C** showing the expression of canonical markers used to define the clusters as scaled values. (**F**) The differentially expressed genes (*P_adj_* < 0.05, Bonferroni-corrected *t* test) between 3 months after dasatinib and diagnosis. The *x* axis denotes the log_2_FC of average expression across single cells. (**G**) The abundances of selected T cell flow cytometry populations as absolute numbers (*n* = 40) following dasatinib treatment. The *P* values were calculated with the Mann-Whitney test. (**H**) UMAP projection of CD8^+^ T cell clusters identified with scRNA-seq (*n* = 4) in Figure 1B. The superimposed line represents the 2 unsupervised predicted maturation trajectories. (**I**) The same as in **H** showing the cell densities at different time points. (**J**) The position of cells in the dasatinib-induced trajectory 2. The *P* values were calculated with the Kruskal-Wallis (left) or Mann-Whitney (right) test. (**K**) The same as in **H** showing the expression of markers used to define the clusters as scaled values. *P < 0.05; **P < 0.01; ****P < 0.0001. Box-and-whisker plots are defined in the Methods.

After the unsupervised immune landscape analyses revealing distinct effects of dasatinib and dasatinib plus IFN-α on the immune repertoire, we studied the effects of dasatinib on the immune cell phenotypes. A thorough analysis of the flow cytometry data revealed that the most significant change after 3 months of dasatinib treatment was early induction of NK and CD8^+^ T cell maturation (Figure 2A, gating strategies in Supplemental Figure 3, A–D).

NK cell maturation proceeds from a CD56^bright^ to CD56^dim^ population, with a simultaneous decrease in cytokine production and increase in cytotoxic potential (20). After dasatinib treatment, we noted a decrease in the CD56^bright^ and cytokine-producing CD27^+^ NK cell (21) populations (both *P_adj_* < 0.01, Benjamini-Hochberg–corrected Mann-Whitney) in the flow cytometry analysis, while the antigen-experienced CD56^dim^CD45RA^+^ NK (*P_adj_* < 0.01), mature CD56^dim^CD57^+^ NK (*P_adj_* < 0.05), and CD56^dim^GZMB^+^ NK (*P_adj_* < 0.001) populations increased significantly (Figure 2B, Supplemental Figure 4A, *P* values for cell type abundances in Supplemental Table 3).

To confirm the early induction of NK cell maturation with dasatinib, we used the pseudotime algorithm Slingshot (22) to order the 3 NK cell clusters identified with scRNA-seq from the most naive to the most mature (see Methods). As expected from the previous scRNA-seq data from healthy donors (23–25), the predicted maturation trajectory recapitulated common NK maturation, as it stemmed from a CD56^bright^ population (cluster 13), progressed to an activated CD56^dim^ population (cluster 3), and ended in the mature CD56^dim^ population (cluster 0, Figure 2C). The trajectory analysis validated the induced maturation, as there was a clear shift from the CD56^bright^ and activated CD56^dim^ population to the terminally mature CD56^dim^ population (Figure 2D). Simultaneously, NK cells seemed to lose their activated function, as they lost genes related to NK cell activation (*FCGR3A* [*CD16*]), effector function, and cancer cell engagement (26) (*PRF1*, *CCL4*, *CD2*); as well as genes related to NF-κB pathway activity (*DUSP1*, *RHO*, *FOSB*) (all *P_adj_* < 0.05, Bonferroni-corrected *t* test, Figure 2, E and F, DEGs in Supplemental Table 2).

Similar to the situation with mature NK cells, dasatinib treatment also increased mature CD8^+^CD57^+^ T cells (*P_adj_* < 0.01, Figure 2G). Like in other trajectory analyses (27, 28), we noted 2 different maturation endpoints for CD8^+^ T cells in the scRNA-seq data stemming from naive CD8^+^ T cells (cluster 8). Trajectory 1 went through the CD8^+^ Temra cluster (cluster 5) and ended in the CD8^+^ Tem phenotype (cluster 1), while trajectory 2 ended in an *IFNG*-producing CD8^+^ Temra cluster (cluster 9, Figure 2H). Interestingly, T cell maturation seemed to shift after dasatinib, as trajectory 1 was more pronounced during diagnosis and trajectory 2 was more prominent after dasatinib treatment. As seen in the trajectory analysis of the NK cells, the CD8^+^ T cells were found in the latter parts of this trajectory (*P* < 0.0001, Kruskal-Wallis, Figure 2J), as the cells had shifted from the homing receptor–positive (ZNF683^+^) Temra cluster 5 to the *IFNG*-producing and IFN-γ–responding Temra population (cluster 9). The changes associated with this transition included downregulation of genes related to T cell naiveness and stem-like properties (*CCR7*, *TCF7*), upregulation of cytotoxic genes (*GZMH*, *GNLY*), different chemokines (*CCL3*, *CCL4*), and *IFNG* and IFN-γ response genes (*IFIT1*, *IFIT2*, *IFIT3*; all *P_adj_* < 0.05, Bonferroni-corrected *t* test, Figure 2K and Supplemental Table 2). Similarly, the DEGs between the 2 trajectories suggested that the dasatinib-associated trajectory is driven by response to IFN-γ, while the other trajectory is driven by NF-κB (Supplemental Figure 5, A–C). In conclusion, dasatinib induced an early maturation of NK and CD8^+^ T cells seen both at the transcriptomic level and in cellular protein level.

### The addition of IFN-α reversed the dasatinib-induced maturation of NK cells and CD8^+^ and CD4^+^ T cells, and restored immunological function.

**Figure 3:**
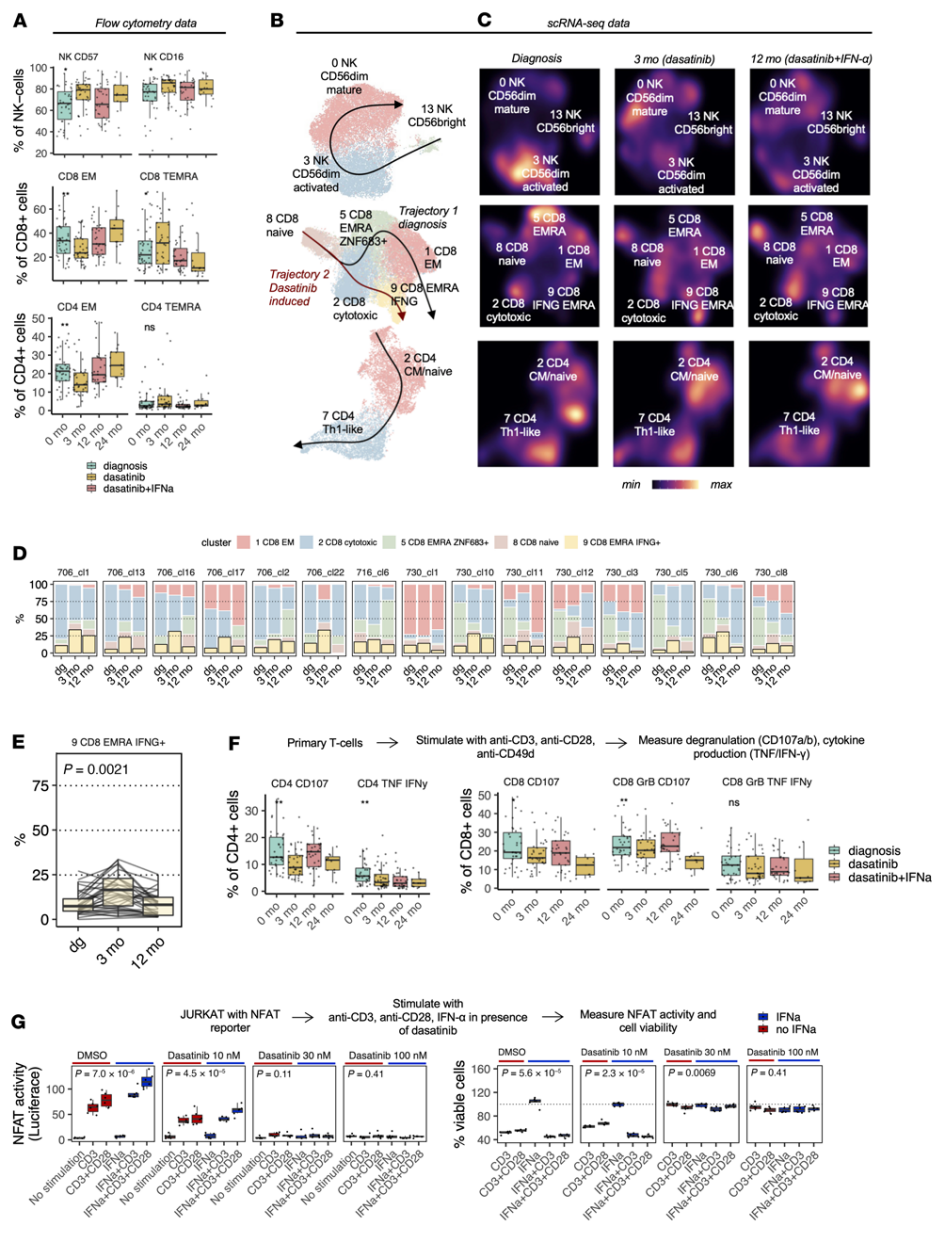
The addition of IFN-α reversed the dasatinib-induced maturation of NK cells and CD8^+^ and CD4^+^ T cells and restored immunological function. (**A**) The abundances of different mature populations of NK cells and CD8^+^ and CD4^+^ T cells at different time points shown as percentages of given parent populations. The *P* values were calculated with the Kruskal-Wallis test. (**B**) UMAP projections of NK cell and CD8^+^ and CD4^+^ T cell clusters identified in Figure 1B, where the superimposed lines represent the predicted maturation trajectories. (**C**) The same as in **B** showing the cell densities at different time points, where the more mature clusters are replaced by immature clusters after the addition of IFN-α to dasatinib. (**D**) The proportion of cells belonging to different clusters in individual CD8^+^ clones, in which the transition toward the *IFNG*-producing Temra cluster (cluster 9) can be seen (15 of 32 [46.88%] clones with at least 5 cells). (**E**) The proportion of CD8^+^ cells belonging to cluster 9 from the 32 clones with at least 5 cells at different time points. The *P* value was calculated with the Mann-Whitney test. (**F**) Cell type abundances of degranulating (CD107^+^) and IFN-γ/TNF-α–producing T cells after being stimulated with anti-CD3, anti-CD28, and anti-CD49d. The *P* values were calculated with the Kruskal-Wallis test. (**G**) The amount of TCR NFAT activity (measured by luciferase) and cell viability (measured by CellTiter Glo assay, normalized to unstimulated wells) after being stimulated with anti-CD3, anti-CD28, and IFN-α in the presence of DMSO or dasatinib. The *P* values were calculated with the 2-sided Kruskal-Wallis test. *P < 0.05; **P < 0.01. NS, not significant. Box-and-whisker plots are defined in the Methods.

Next, we focused on the changes in immune cell phenotypes induced by the addition of IFN-α to dasatinib therapy. scRNA-seq and flow cytometry data revealed that the combination therapy partly reversed the dasatinib-induced maturation of NK cells and CD8^+^ and CD4^+^ T cells. The number of mature CD56^dim^CD57^+^ and CD56^dim^CD16^+^ NK cell phenotypes reduced after combination therapy (*P_adj_* < 0.05, Benjamini-Hochberg–corrected Kruskal-Wallis, Figure 3A, P values for cell type abundances in Supplemental Table 3). In the scRNA-seq data, this was observed as a shift back in the pseudotime as the number of mature CD56^dim^ cells (cluster 0) decreased (Figure 3, B and C). Similarly, the proportion of CD8^+^ Temra cells decreased (*P_adj_* < 0.05) and CD8^+^ Tem cells increased (*P_adj_* < 0.01) (Figure 3, A and B). In addition, the proportion of CD4^+^ Tem cells increased (*P_adj_* < 0.05) and there was a trend toward decreased CD4^+^ Temra cells (Figure 3A). The maturation trajectory in CD4^+^ T cells went from naive/central memory (Tcm/n, cluster 2) to Th1-like (cluster 7), and the addition of IFN-α to dasatinib resulted in a shift back to more immature CD4^+^ Tcm/n cells (Figure 3, B and C).

Next, we performed in-depth scRNA-seq plus scTCRαβ-seq analysis at a T cell clonotype level to study whether we could also reproduce the trajectories with individual T cell clones in vivo. We selected T cell clones that had at least 5 cells in the scRNA-seq plus scTCRαβ-seq data (32 clones, all CD8^+^). Fifteen (46.68%) CD8^+^ T cell clones behaved similarly to the total CD8^+^ T cell population, and the proportion of *IFNG*-producing Temra cells (cluster 9) increased following dasatinib and decreased following the addition of IFN-α (*P* < 0.01, two-sided Mann-Whitney, Figure 3, D and E), reproducing the CD8^+^ T cell trajectory at the clonotype level.

To address how these findings translate to the functional capabilities of the lymphocyte populations, we performed (a) ex vivo degranulation and (b) cytokine production analysis on primary samples from different time points, as well as (c) a TCR activity assay with a Jurkat reporter cell line.

For the ex vivo analyses, the CD8^+^ and CD4^+^ T cells were stimulated with anti-CD3, anti-CD28, and anti-CD49d antibodies and NK cells with CML cell line K562 (gating strategies in Supplemental Figure 6, A–C). The production of TNF-α and IFN-γ was higher at diagnosis than during treatment in CD8^+^ (*P* < 0.01, Kruskal-Wallis) and CD4^+^ T cells (*P* < 0.01, Kruskal-Wallis) (Figure 3F and Supplemental Table 3). After 3 months of dasatinib treatment, the degranulation responses (CD107^+^) diminished in both CD8^+^ (*P* < 0.05, Mann-Whitney) and CD4^+^ T cells (*P* < 0.01, Figure 3F), potentially highlighting the inhibitory effect of dasatinib on cellular functional capabilities. After the addition of IFN-α to dasatinib, the degranulation of both CD8^+^ and CD4^+^ T cells markedly improved compared with dasatinib-only values (both *P* < 0.05) and were at the same level as at diagnosis (both insignificant). At 24 months, after the discontinuation of IFN-α therapy, the degranulation responses of T cells were lower than at 12 months during combination therapy (Figure 3F). There was a similar, yet statistically insignificant, trend with NK cells, as degranulation responses decreased with dasatinib treatment and increased after the addition of IFN-α (Supplemental Figure 6D).

To measure how dasatinib and IFN-α affect T cell activation, we used a Jurkat TCR reporter cell line with a luciferase reporter under the control of an *NFAT* response element. TCR (NFAT) activity and cell viability were measured following CD3, CD28, and IFN-α stimulation with and without different dasatinib concentrations (10 nM, 30 nM, and 100 nM; see Methods). Dasatinib suppressed both anti-CD3/anti-CD28–mediated and IFN-α–mediated TCR activation in a dose-dependent manner, and its full inhibitory effect was already seen at the 30 nM concentration (Figure 3G). Interestingly, IFN-α was found to provide a strong costimulatory signal for TCR activation, and its effects were comparable to those seen with CD28 costimulation (Figure 3G). Strong CD3 stimulation caused activation-induced cell death (AICD), which was measured as decreased viability of Jurkat cells at 24 hours. Markedly, dasatinib protected cells from AICD in a dose-dependent manner, resulting in similar reporter cell viability in CD3-stimulated and unstimulated control cells (Figure 3G). Together, these findings suggest that dasatinib may induce accumulation of terminally differentiated NK and T cells with diminished functionality, as cells do not die through normal AICD.

### Dasatinib plus IFN-α widens the predicted epitope landscape of CD8^+^ T cells and expands anti-CMV T cells.

**Figure 4:**
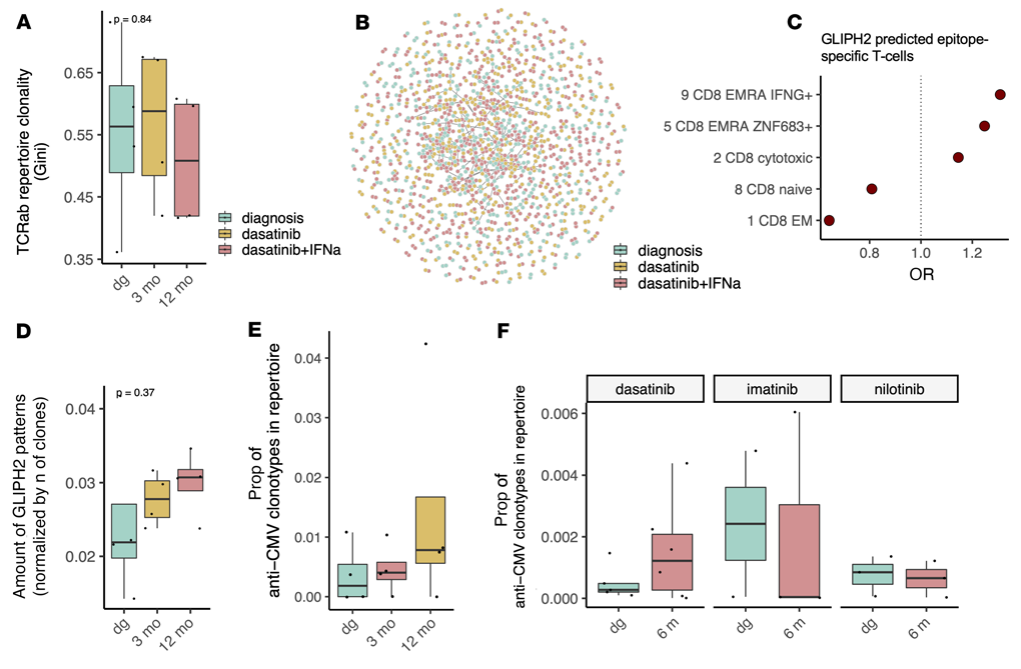
The addition of IFN-α widens the predicted epitope landscape of CD8^+^ T cells and reactivates anti-CMV T cells. (**A**) The T cell receptor (TCRαβ) repertoire clonality from the scTCRαβ-seq data (*n* = 4) measured by Gini index, where higher values indicate more clonal, i.e., less diverse, samples. The *P* value was calculated with the Kruskal-Wallis test. (**B**) Network plot showing the putative epitope-specific TCR groups predicted by GLIPH2. Each node is a TCR, and the edges denote a GLIPH2-predicted shared epitope-specific target. (**C**) The odds ratios (ORs) for the phenotypes for GLIPH2-predicted epitope-specific CD8^+^ T cells identified in the scRNA-seq and scTCRαβ-seq data. (**D**) The number of epitope-specific groups predicted by GLIPH2 normalized by the number of different clonotypes in each sample. The *P* value was calculated with the Kruskal-Wallis test. (**E**) The proportions of TCRGP-predicted cytomegalovirus-specific (CMV-specific) CD8^+^ T cells from the scTCRαβ-seq data. (**F**) The proportions of TCRGP-predicted CMV-specific T cells from bulk TCRβ-seq data from the peripheral blood from patients treated with dasatinib (*n* = 6), imatinib (*n* = 4), or nilotinib (*n* = 3). Box-and-whisker plots are defined in the Methods.

After discovering the beneficial effect of the added IFN-α on the functional properties of T cells, we asked whether this affects the TCR repertoire diversity. Due to the low number of samples in the scTCRαβ-seq data, no significant changes in the clonality were observed, although dasatinib seemed to increase the clonality, and this was partly reversed by the addition of IFN-α (Figure 4A).

As previous studies have shown that IFN-α induces T cell antigen recognition (29), we hypothesized that IFN-α could also broaden the epitope landscape of T cells. To study this, we used GLIPH2 (30) to sort CD8^+^ TCRs into potential epitope-specific groups based on the TCR similarity (for performance of GLIPH2 in epitope-specific data sets, see Supplemental Figure 7 and Methods). In total, GLIPH2 predicted 115 epitope-specific groups for CD8^+^ T cells (Figure 4B, GLIPH2 results in Supplemental Table 4). The CD8^+^ T cells clustered in epitope-specific groups were preferentially of the CD8^+^ Temra phenotype (clusters 9 and 5, Figure 4C), providing an internal validation for the enrichment for epitope-specific T cells. The amount of these epitope-specific groups increased following both dasatinib and dasatinib plus IFN-α combination therapy (Figure 4D), but this was not statistically significant.

As GLIPH2 is an unsupervised algorithm, it is unaware of the epitopes for the clusters it predicts. Therefore, we used TCRGP (31) — our recent machine-learning classifier — to evaluate the probabilities of TCRs to recognize previously known epitopes from common viral infections, including cytomegalovirus (CMV), Epstein-Barr virus (EBV), influenza A virus, and herpes simplex virus 2 (HSV2) (for performance of TCRGP in epitope-specific data sets, see Supplemental Figure 8 and Methods). TCRGP revealed that the most common target was CMV epitope pp65_NLV_ (1.67% [10/600] of clonotypes with at least 2 cells, TCRGP results in Supplemental Table 5). Interestingly, the proportion of predicted anti-CMV clonotypes increased during dasatinib treatment (Figure 4E), although CMV viremia or CMV manifestations were not reported in any of the patients.

We explored the expansion of CMV-related clones further by performing bulk TCRβ-seq on bone marrow–derived mononuclear cell samples from newly diagnosed CP-CML patients before and after 6 months of treatment with dasatinib (*n* = 6), imatinib (*n* = 4), or nilotinib (*n* = 3) from the previous first-line NordCML006 (dasatinib or imatinib) (32, 33) and ENEST1st (nilotinib) (34–36) trials (patient details in Supplemental Table 1). Dasatinib was the only TKI that increased the abundance of predicted anti-CMV T cell clonotypes (Figure 4F), but this finding was insignificant possibly due to the low number of samples.

### The addition of IFN-α increases the amount of costimulatory cell-cell interactions via B cells and monocytes.

**Figure 5:**
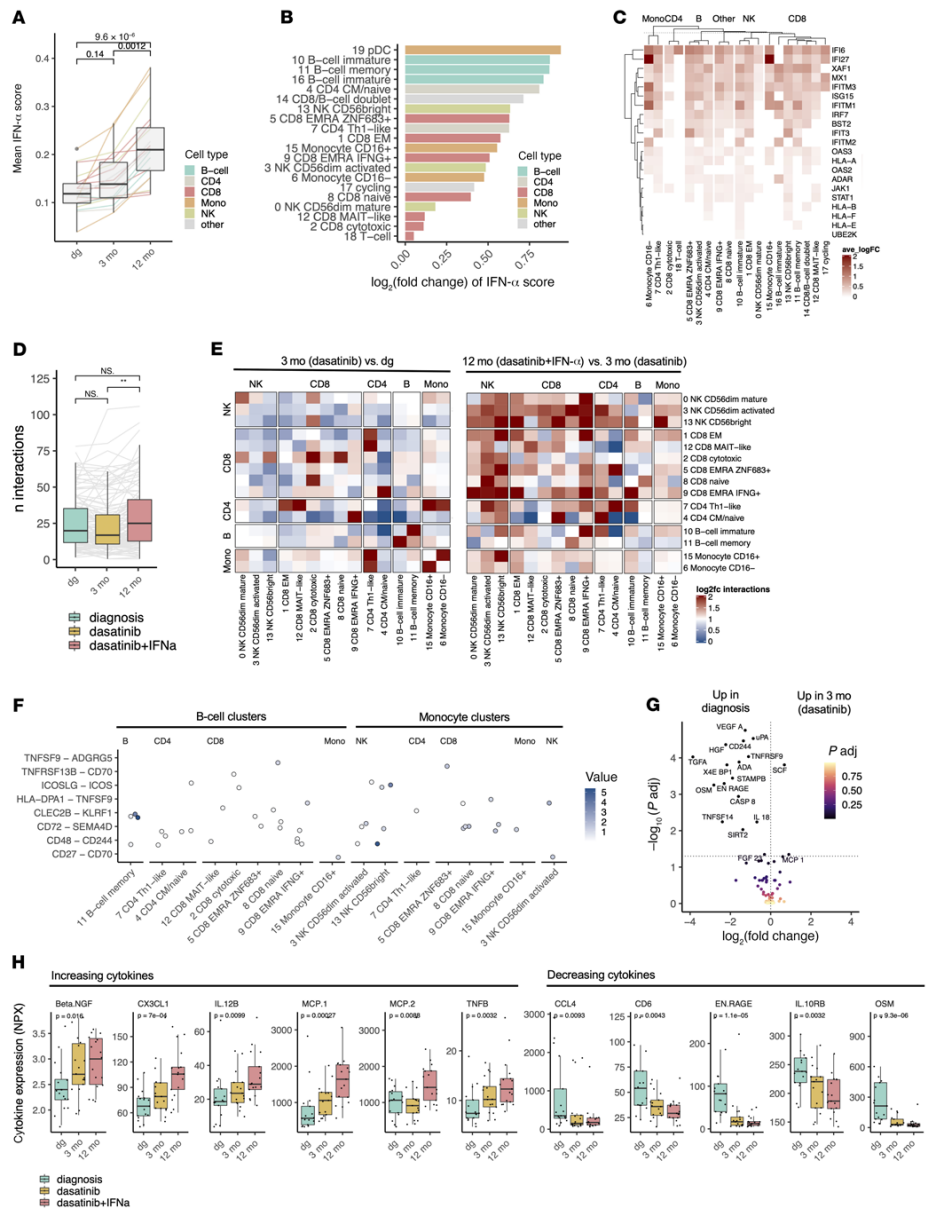
The addition of IFN-α increases the amount of stimulatory cell-cell interactions via B cells and monocytes. (**A**) The median IFN-α response score in different clusters at different time points identified with scRNA-seq. The *P* values were calculated with the Kruskal-Wallis test. (**B**) The log_2_-transformed fold change (log_2_FC) of the median IFN-α response score in different clusters between the 12-month (dasatinib+IFN-α) and 3-month (dasatinib) time points. (**C**) Heatmap showing the differentially expressed genes (*P_adj_* < 0.05, Bonferroni-corrected *t* test) related to IFN-α response between the 12-month (dasatinib + IFN-α) and 3-month (dasatinib) time points. (**D**) The numbers of ligand-receptor interactions predicted by CellPhoneDB at different time points. **P = 0.011 by the Kruskal-Wallis test. (**E**) Heatmap showing the log_2_FC of the change in the numbers of significant (*P_adj_* < 0.05, Benjamini-Hochberg–corrected CellPhoneDB test) ligand-receptor interactions between 3 months (dasatinib) and diagnosis and between the 12-month (dasatinib + IFN-α) and 3-month (dasatinib) time points. (**F**) The costimulatory immune interactions after the addition of IFN-α to dasatinib (12 months) as predicted by CellPhoneDB in 4 different B cell clusters and 3 different monocyte clusters. (**G**) Volcano plot showing the differentially expressed (*P_adj_* < 0.05, Benjamini-Hochberg–corrected Mann-Whitney) cytokines between the 3-month (dasatinib) and diagnosis time points. The *x* axis denotes the log_2_FC of median cytokine expression, where higher values denote upregulation at the 3-month time point. (**H**) The expression of different statistically differentially expressed (*P_adj_* < 0.05, Benjamini-Hochberg–corrected Kruskal-Wallis) cytokines. The *P* values were calculated with the Kruskal-Wallis test. NPX, normalized plasma expression. Box-and-whisker plots are defined in the Methods.

To study the transcriptional changes induced by dasatinib plus IFN-α combination treatment, we performed DEG and pathway analyses on different immune subpopulations. As expected, scRNA-seq data revealed that the addition of IFN-α resulted in significant upregulation of IFN-α response genes as measured by an IFN-α response module score (Methods, *P* < 0.01, Mann-Whitney, Figure 5A). By calculating the fold change between, before, and after the addition of IFN-α to dasatinib, we noted that the most IFN-α–responding clusters were plasmacytoid dendritic cells (pDCs), a major physiological source of IFN-α (37) and previously associated with treatment-free remission in CP-CML (38, 39), and 3 different B cell clusters (Figure 5B). The upregulated IFN-α response genes include genes related to the JAK/STAT pathway (*JAK1*, *STAT1*), IFN-γ response genes (*IRF7*, *IFIT3*), and importantly, HLA class I genes (*HLA-B*, *HLA-E*, *HLA-F*) (Figure 5C, DEGs in Supplemental Table 2).

As the different class I HLAs were upregulated following the addition of IFN-α, we next asked whether the addition of IFN-α could increase the number of cell-cell communications. Ligand-receptor interaction analysis with CellPhoneDB (40) demonstrated that the amount of cellular communication increased significantly after the addition of IFN-α to dasatinib (*P* < 0.01, Mann-Whitney test, Figure 5D, immune interactions in Supplemental Table 6). The most changes in the number of ligand-receptor interactions were observed in the crosstalk between NK cells and CD8^+^ T cells that are important for the anticancer activity of NK cells (26), but also in, for example, monocyte–NK cell, monocyte–CD8^+^ T cell, B cell–NK cell, and B cell–CD8^+^ T cell interactions (Figure 5E). Most interactions between B cells, monocytes, and the cytotoxic lymphocytes were costimulatory, including CD72-SEMA4D known to enhance IFN-γ secretion of NK cells (41), CELC2B-KLRF1 known to promote cytolysis (42), and ICOSLG-ICOS involved in the adaptive CD4^+^ T cell responses (43) (Figure 5F). Additionally, many inhibitory receptor-ligand interactions were downregulated, such as *TNFRSF14* (HVEM) and its associated molecules (*MIF*, *CD160*, *BTLA*) (44) and *LGALS9* (galectin 9)–*HAVCR2* (TIM-3) (45).

To study the cellular communication further, we profiled the levels of 50 plasma proteins with a multiplexed approach from 3 different time points (*n* = 17, in total 80 samples). After 3 months of dasatinib, the levels of several cytokines decreased, including VEGF-A, TNFRSF9, and TGF-α (all *P_adj_* < 0.001, Benjamini-Hochberg–corrected Mann-Whitney, Figure 5G, P values in Supplemental Table 3). However, the addition of IFN-α increased the levels of multiple cytokines, including BNGF, CX3CL1, IL-12B, MCP1, MCP2, and TNF-β (all *P_adj_* < 0.05, Benjamini-Hochberg–corrected Kruskal-Wallis, Figure 5H and Supplemental Figure 9A, *P* values in Supplemental Table 3). Simultaneously, the levels of immune inhibitory cytokines were decreased, including IL-10RB (46) and OSM (47) (Figure 5H). Together, these data highlight the potentially beneficial role of IFN-α in the orchestrated immune activation following dasatinib monotherapy.

### Immunological biomarkers associated with treatment response and adverse effects.

**Figure 6:**
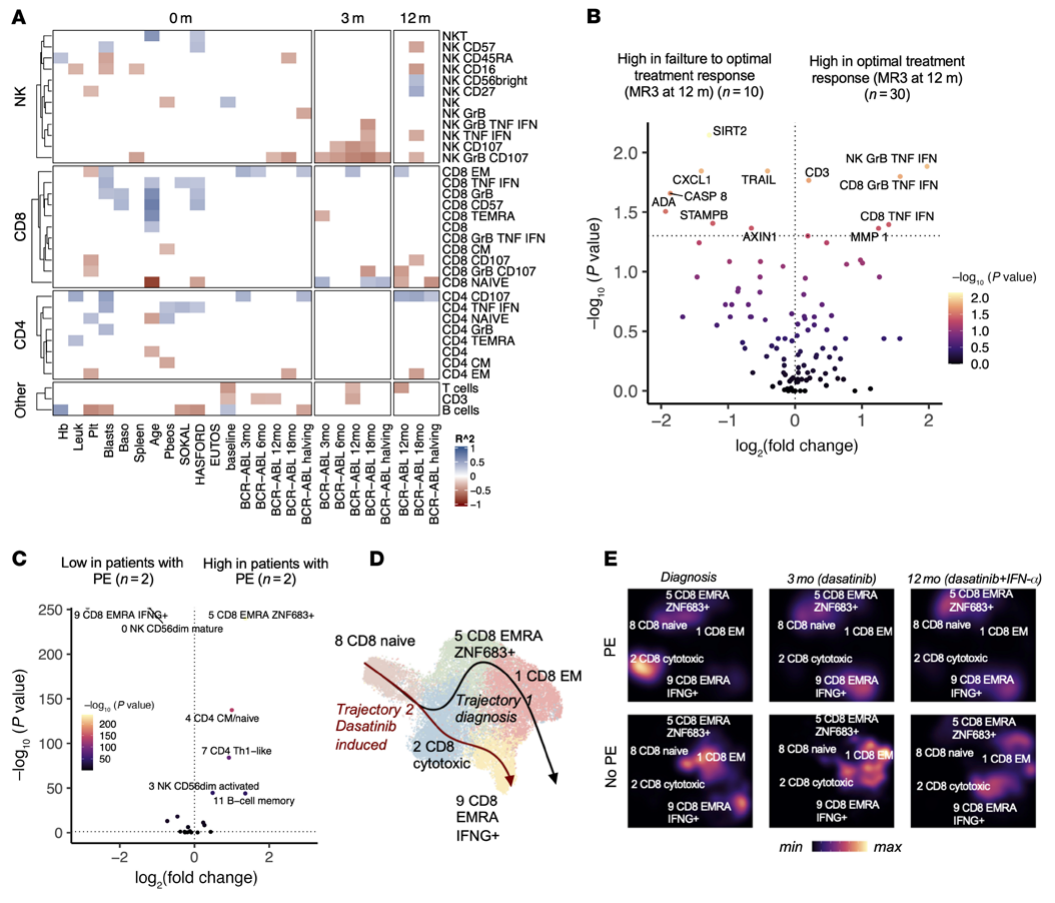
Immunologic biomarkers associated with treatment response and adverse effects. (**A**) Heatmap showing the *R*^2^ values from Spearman’s rank correlation analysis of flow cytometry populations with the clinical parameters. Only correlations with *P* < 0.1 are shown. (**B**) Volcano plot showing the differentially abundant (*P* < 0.05, Mann-Whitney test) immune cell populations in the flow cytometry cohort and the differentially expressed cytokines between the patients with early treatment success (defined as <0.1% *BCR-ABL1^IS^* levels at 12 months) to patients failing to reach this milestone. The *x* axis denotes the log_2_-transformed fold change (log_2_FC) of median population abundance in clusters, where higher values denote upregulation in patients with optimal treatment response. (**C**) Volcano plot showing the differentially abundant (*P_adj_* < 0.05, Benjamini-Hochberg–corrected Fisher’s 2-sided exact test) scRNA-seq populations between patients with and without dasatinib-associated pleural effusion (PE). The *x* axis denotes the log_2_FC of median population abundance in clusters, where higher values denote upregulation in patients with PE. (**D**) UMAP projections of CD8^+^ T cell clusters identified in Figure 1B, where the superimposed lines represent the predicted maturation trajectories. (**E**) The same as in **D** showing the cell densities at different time points in patients with (*n* = 2) or without (*n* = 2) dasatinib-associated PE.

Finally, to translate these detailed immunological findings to the clinic, we correlated our immune monitoring results with clinically important covariates and endpoints (clinical data in Supplemental Table 1, time to reach clinical responses in Supplemental Figure 10A). As we noted that IFN-α increased the proportion of degranulating lymphocytes, it was interesting to discover that they were associated with better therapy responses. The patients who had more degranulating and cytokine-producing NK cells (CD107^+^ NK, CD107^+^GZMB^+^ NK, TNF^+^ NK, and TNF-α^+^IFN-γ^+^ NK) after 3 months of therapy with dasatinib had lower *BCR-ABL1* transcript levels at follow-up time points (Figure 6A, correlation *P* values in Supplemental Table 7). When further comparing the patients who had optimal treatment response (defined as <0.1% *BCR-ABL1^IS^* levels [MMR3] at 12 months) (*n* = 30) to patients failing to reach this milestone (*n* = 10), the most specific immunological alteration was the amount of TNF-α/IFN-γ–producing NK and CD8^+^ T cells at baseline (*P* < 0.05, *P_adj_* > 0.05, Figure 6B, at 3 months and 12 months see Supplemental Figure 10, B and C). Instead, the expression of multiple cytokines, including SIRT1 and CXCL1, at the baseline was associated with treatment failure (Figure 6B and Supplemental Figure 11A).

As the clinical trials suggested that dasatinib plus IFN-α resulted in fewer dasatinib-associated PEs, we tried to find immunological mechanisms that could explain the lower prevalence of this common side effect. The median age of patients with PE or pulmonary arterial hypertension (PAH) (*n* = 5) was higher than in patients without PE/PAH (*n* = 35) (*P* < 0.05, two-sided Mann-Whitney). The immunological parameters correlated with higher age were increased amounts of mature T cells, including CD8^+^ Temra, CD8^+^, CD57^+^, and CD8^+^GZMB^+^ T cells (Figure 6A and Supplemental Figure 11B). Similarly, in the scRNA-seq data, patients with PE (*n* = 2) in comparison with patients without PE/PAH (*n* = 2) had elevated amounts of mature cells. Patients with PE/PAH had more CD8^+^ Temra cells expressing homing receptor *ZNF683* transcripts (cluster 5) throughout the treatment (*P* < 0.0001, Fisher’s 2-sided test) and fewer *IFNG*-producing CD8^+^ Temra cells (cluster 9) than patients without PE/PAH (Figure 6C and Supplemental Table 3). The *IFNG*-producing cells were the endpoint for the dasatinib-associated maturation trajectory (Figure 6D), indicating that in patients with PE/PAH, CD8^+^ T cell maturation did not end as expected. Overall, these results highlight how changes in the immune profile can be linked to therapeutic outcomes in patients with CP-CML.

## Discussion

The hypothesis of immunological surveillance in CML has garnered increased interest in the quest to increase the rate of treatment-free remission (4, 48). Here, we show that the combination of 2 immunomodulatory treatments — dasatinib and IFN-α — induces unique changes in both innate and adaptive immune systems and show how they are linked to clinical outcomes in patients with CP-CML.

NK cells, an integral part of innate immunity, have special importance in CML, as recent studies have shown that the high frequency of NK cells is associated with deep molecular response (49) and successful TKI (imatinib and dasatinib) treatment discontinuation (50–52). Hence, it was interesting to note that dasatinib treatment increased both the frequency and absolute numbers of NK cells. However, the largest increase was noted in the terminally differentiated mature CD56^dim^ NK cells with decreased degranulation responses, and scRNA-seq suggested that the expression of genes related to NK cell cytotoxicity — such as *PRF1*, *RHOB*, and *GZMA* — was decreased. Intriguingly, the addition of IFN-α reversed the transcriptional changes and this was noted as improved ex vivo degranulation responses of NK cells. Also, in a recent study by Alves et al., TKI plus IFN-α–treated patients had increased numbers of CD56^bright^ NK cells (53). The NK cell degranulation also correlated with lower *BCR-ABL1^IS^* levels at the follow-up time points (3 and 12 months), which helped the patients to reach optimal treatment response at the 12-month time point. The improved degranulation responses could also partly relate to lower tumor load, as previous studies have shown that deep molecular remission with TKIs correlates with increased NK counts, cytotoxicity, and CD8^+^ T cells (49–54). However, the analysis of 24-month samples (9 months after IFN-α discontinuation at 15 months) suggested that the degranulation responses were lower during continued dasatinib monotherapy than at 12 months when combination therapy was used. This highlights the beneficial effect of IFN-α treatment.

Like NK cells, T cells also increased in number, and their phenotypes also changed to terminally mature cells during dasatinib treatment. The CD8^+^ and CD4^+^ T cells also partially lost their degranulation ability and CD4^+^ T cells lost cytokine secretion. This is in agreement with recent data suggesting that dasatinib can act as a reversible on/off switch for CAR T cells (55, 56). Interestingly, our data with a Jurkat TCR reporter cell line suggest that dasatinib not only prevents TCR signaling, but it can also spare cells from strong-stimulation-induced cell death via inhibiting TCR/NFAT(-FASL) activation, and this may lead to the accumulation of Temra cells, as they are not dying through normal AICD. This could partially explain the previously noted discrepancy of in vivo and in vitro immunomodulatory effects of dasatinib. Interestingly, we also found that IFN-α can induce a strong costimulatory signal for TCR activation, which is consistent with the observed improved functionality of T cells in patients after the addition of IFN-α.

The dasatinib-induced T cell maturation and inverse effects of IFN-α were also noted at a single T cell clonotype level. The increase in mature T cells was accompanied with an increase in predicted epitope-specific T cells studied with the unsupervised GLIPH2 algorithm measuring similarities of TCRs (30). With the supervised TCRGP algorithm (31), where we used available tetramer-specific-TCR data on common viruses, we noted that the proportion of T cells that were predicted to target the CMV pp65 epitope seemed to increase following dasatinib, and the bulk TCRβ-seq from samples from the previous first-line NordCML006 (dasatinib or imatinib) (32, 33) and ENEST1st (nilotinib) (34–36) trials confirmed that CMV-specific T cell clonotypes expanded only during dasatinib treatment. This is of special interest, as reactivations of latent CMV have been associated with dasatinib treatment, especially in patients with multiple prior treatment lines (7). Clinical CMV reactivations were not observed in our patient cohort, but no CMV PCR monitoring was performed. Therefore, the possibility of subclinical reactivations cannot be ruled out. Future studies with TCRGP armed with training data of TCRs against leukemia-associated antigens, such as WT1 and PR1, could provide crucial information on the antitumor immunity in patients with CML treated with different therapies.

Besides the NK and T cells, B cells are noted to be affected by off-target TKI responses, resulting in decreased immunoglobulin levels, loss of memory status, and downregulation of key kinases implicated in B cells (49, 57, 58). Hence, it was interesting to note that different B cell classes benefited from the addition of IFN-α, with an increased amount of costimulatory interactions and cytokines. Cytokines that increased during the treatment — CX3CL1, MCP1 (CCL2), and MCP2 (CCL8) — are chemoattractant for leukocytes, while TNF-β regulates leukocyte proliferation, differentiation, and survival. Many of the treatment failure–associated cytokines (e.g., SIRT1 and CXCL1) could support leukemia stem cell proliferation and survival (59).

The previously published clinical results from the NordCML007 study showed a lower initial incidence of PE than expected (1-year incidence of 1 out of 40 patients treated with the combination) (10, 11), which is in line with data from the French study (1 out of 80) (18). In comparison, the first-line dasatinib monotherapy study, DASISION, showed a 1-year incidence of PE of 10% and a 5-year incidence of 28% (2). Older age is a recognized risk factor for PE (60, 61), and this is in line with our observation showing that patients with PE had an increased proportion of highly differentiated CD8^+^ T cells, which have been associated with older age (62). It has also been shown that increased amounts of large granular lymphocytes (LGLs) are observed in patients with dasatinib-induced PE (5, 63), correlating well with the highly differentiated cytotoxic T cell phenotype in our data. In addition, as the increased amounts of cytotoxic LGLs are shown to reside especially in pleural fluid samples of patients with PE (5), it was intriguing to see that patients with PE had elevated amounts of CD8^+^ Temra cells expressing the homing receptor ZNF683. The addition of IFN-α resulted in a significant decrease in the frequency of mature CD8^+^ T cells, but the mechanism(s) of how these T cells could be associated with the PE/PAH occurrence needs further elucidation. Dasatinib may promote the extravasation of lymphocytes to the pleural space, as it inhibits SRC family kinases that are known to regulate adhesive interactions of epithelial cells (64, 65).

Taken together, we observed that IFN-α and dasatinib treatments have differential effects on the immune system. It could be speculated whether dasatinib may drive immunity too excessively into an oligoclonal cytotoxic effector cell state, whereas the combination with IFN-α may widen the immune repertoire and increase the interaction of different immune cells. Similar effects have been observed in solid tumor trials where anti–CTLA-4 has been combined with anti–PD-1, resulting in a widened immune repertoire and reactivation of the putative tumor-reactive, terminally differentiated memory T cells with clinical benefit (66). Ongoing clinical trials, such as the German Tiger trial (NCT01657604) (67), will address whether the beneficial effects of low-dose IFN-α will also translate into improved long-term clinical outcomes, especially as increased rates of treatment-free remission.

## Methods

### Patients and samples.

Forty newly diagnosed CML patients participated in the NordCML007 clinical trial (NCT01725204) (10, 11). Patients were treated with 100 mg dasatinib q.d. and after 3 months IFN-α treatment was added (first 3 months 15 μg/week, then 25 μg/week of pegylated IFN-α). After 12 months of combination treatment, patients resumed dasatinib monotherapy. In this immunological substudy, PB samples were collected at diagnosis and 3, 12, and 24 months after the start of therapy. PB samples were collected before the daily dasatinib dose.

PB mononuclear cells (PBMCs) were isolated with Ficoll-Paque (GE Healthcare) density gradient centrifugation. Fresh PBMCs were used for immunophenotyping of T and NK cells. The remaining PBMCs were stored in liquid nitrogen and plasma was frozen at –70°C. All the experiments were performed once per patient sample.

### NK and T cell phenotyping and analysis.

T and NK cell markers were stained with conjugated antibodies. For staining of T cells, the following antibodies were used: CD45-APC H7 (2:100; BD, clone 2D1, cat. 641417), CD3-APC/PeCy7 (0.5:100/5:100; BD, cat. 561810/557851), CD4-PerCP (4:100; BD, cat. 553052), CCR7-PE (1:10; Biotechne, cat. FAB197P-100), CD45RA–Alexa Fluor 700 (5:100; BD, cat. 560673), CD27-V500 (1:100; BD, cat. 561222), and CD57-FITC (1:10; BD, cat. 555619). For staining of NK cells, the following antibodies were used: CD45-APC H7, CD19/CD14–Pacific Blue (5:100/5:100; Invitrogen, cat. MHCD1617/MHCD1428), CD3-APC/PeCy7, CD56-PE (2:100; BD, cat. 345812), CD45RA–Alexa Fluor 700, CD27-V500, CD57-FITC, and CD16–Texas Red (4:100; Invitrogen, cat. MHCD1617). The analyzed populations are defined in Supplemental Table 3. The absolute numbers of populations were determined by multiplying the flow cytometry populations with the differential counts obtained from the electronic health records. The gating strategies can be seen in Supplemental Figures 3 and 5.

The identified cell population abundances and absolute numbers were first visualized with PCA to understand overall trends and detect possible outliers. The PCA was performed in base R (4.0.0) (https://www.r-project.org/) with the “prcomp” function, where scaling was used. The PCA was visualized in the first 20 dimensions, and the weights for the components were also analyzed. Afterwards, the cell populations were used in statistical testing to detect differences between different treatment time points. The statistical testing was performed with nonparametric tests (Mann-Whitney for 2 groups, Kruskal-Wallis for more than 2 groups), and the *P* values were further corrected with the Benjamini-Hochberg method in base R (4.0.0).

### Plasma cytokine analysis.

A multiplexed cytokine and growth factor panel including 50 proteins was performed using 78 plasma samples from 17 patients (samples collected at diagnosis, 3 months, and 12 months) (Proseek Multiplex Inflammation I^96×96^, Olink). The first 17 patients included in the study were selected for the analysis. Similar to flow cytometry data analysis, the received normalized plasma expression levels provided by Olink for cytokines were first analyzed with PCA with the “prcomp” function in base R (4.0.0), after which statistical testing was performed with nonparametric tests (Mann-Whitney for 2 groups, Kruskal-Wallis for more than 2 groups), and the *P* values were further corrected with the Benjamini-Hochberg method in base R (4.0.0).

### scRNA-seq and scTCRαβ-seq.

Single cells were partitioned using a Chromium Controller (10× Genomics) and scRNA-seq and scTCRαβ-seq libraries were prepared using a Chromium Single-Cell 5′ Library & Gel Bead Kit (10× Genomics), as per the manufacturer’s instructions (CG000086 Rev D), with a target of 7,500 to 25,000 cells from each sample and as previously published by us (68). The cells were suspended in 0.04% BSA in PBS and were loaded on the Chromium Single-Cell A Chip. Full-length cDNA was amplified using 14 cycles of PCR (Veriti, Applied Biosystems). TCR cDNA was further amplified in a heminested PCR reaction using a Chromium Single-Cell Human T Cell V(D)J Enrichment Kit (10× Genomics). The total cDNA and the TCR-enriched cDNA were subjected to fragmentation, end-repair and A-tailing, adaptor ligation, and sample index PCR (14 and 9 cycles, respectively). The gene expression libraries were sequenced using an Illumina NovaSeq S1 flow cell with the following read length configuration: Read1 = 26, i7 = 8, i5 = 0, Read2 = 91. The TCR-enriched libraries were sequenced using an Illumina HiSeq 2500 in Rapid Run mode with the following read length configuration: Read1 = 150, i7 = 8, i5 = 0, Read2 = 150. The raw data were processed using Cell Ranger 3.0.0 (https://support.10xgenomics.com/single-cell-gene-expression/software/downloads/latest) with GRCh38 as the reference genome with default parameters.

### scRNA-seq and scTCRαβ-seq data analysis.

All cells were subject to quality control. Cells with high amounts of mitochondrial transcripts (>15% of all UMI counts) or ribosomal transcripts (>50%), cells with less than 100 genes or more than 4,500 genes expressed, cells expressing low or high (<25% or >60%) numbers of housekeeping genes, or cells with low or high read depth (<500 or >30,000) were excluded from the analyses.

To overcome batch effects, we used a recently described probabilistic framework to overcome different nuisance factors of variation in an unsupervised manner with deep generative modeling with scVI (0.5.0) (19). Briefly, the transcriptome of each cell is encoded through a nonlinear transformation into a low-dimensional, batch-corrected latent embedding as per vignette with default parameters. The latent embedding was then used for graph-based clustering implemented in Seurat (3.0.0) (https://satijalab.org/seurat/articles/install.html) and UMAP-dimensionality reduction (69). Clusters were annotated by analysis of canonical markers, DEGs, relationship to other clusters, signature scores, TCR repertoire, and automated cell type annotation with SingleR (70) (1.2.4) based on sorted immune subsets with default parameters.

Differential expression analyses were performed with the FindMarkers function implemented in Seurat (3.0.0) based on the *t* test, and the *P* values were further corrected with Bonferroni’s method. Pseudotime analyses were done with Slingshot (22) in unsupervised mode on precalculated UMAP coordinates from latent dimensions with default parameters. The DEGs between the 2 CD8^+^ T cell trajectories were analyzed with the “diffEndTest” function in tradeSeq (71) (1.6.0) with default parameters. Genes were annotated as differentially expressed if the *P_adj_* was less than 0.05 and the average fold-change was greater than 0.10. The DEGs were further evaluated with enrichment analysis, which was performed with hypergeometric testing implemented in the clusterProfiler (72) (4.0.5) R package on HALLMARK, GO, KEGG, and REACTOME categories.

Different scores were calculated with Seurat’s AddModuleScore-function, which is an implementation of the method suggested by Tirosh et al. (73). Briefly, it calculates the average expression levels of selected genes at a single-cell level from which is then subtracted a similarly counted expression of randomly selected control feature sets. The cytotoxicity score was calculated as described with genes suggested by Dufva, Pölönen, et al. (74), including *GZMA*, *GZMB*, *GZMH*, *PRF1*, and *GNLY*, while the exhaustion score was calculated with *CTLA4*, *PDCD1*, *LAG3*, *HAVCR2*, and *TIGIT*, and HLA class II score with *HLA-DRA*, *HLA-DRB5*, *HLA-DRB1*, *HLA-DQA1*, *HLA-DQB1*, *HLA-DQB1-AS1*, *HLA-DQA2*, *HLA-DQB2*, *HLA-DOB*, *HLA-DMB*, *HLA-DMA*, *HLA-DOA*, *HLA-DPA1*, and *HLA-DPB1*. The IFN-α response score was calculated with genes gathered from HALLMARK IFN-α response pathway genes, including *ADAR*, *B2M*, *BATF2*, *BST2*, *C1S*, *CASP1*, *CASP8*, *CCRL2*, *CD47*, *CD74*, *CMPK2*, *CMTR1*, *CNP*, *CSF1*, *CXCL10*, *CXCL11*, *DDX60*, *DHX58*, *EIF2AK2*, *ELF1*, *EPSTI1*, *GBP2*, *GBP4*, *GMPR*, *HELZ2*, *HERC6*, *HLA-C*, *IFI27*, *IFI30*, *IFI35*, *IFI44*, *IFI44L*, *IFIH1*, *IFIT2*, *IFIT3*, *IFITM1*, *IFITM2*, *IFITM3*, *IL15*, *IL4R*, *IL7*, *IRF1*, *IRF2*, *IRF7*, *IRF9*, *ISG15*, *ISG20*, *LAMP3*, *LAP3*, *LGALS3BP*, *LPAR6*, *LY6E*, *MOV10*, *MVB12A*, *MX1*, *NCOA7*, *NMI*, *NUB1*, *OAS1*, *OASL*, *OGFR*, *PARP12*, *PARP14*, *PARP9*, *PLSCR1*, *PNPT1*, *PROCR*, *PSMA3*, *PSMB8*, *PSMB9*, *PSME1*, *PSME2*, *RIPK2*, *RNF31*, *RSAD2*, *RTP4*, *SAMD9*, *SAMD9L*, *SELL*, *SLC25A28*, *SP110*, *STAT2*, *TAP1*, *TDRD7*, *TENT5A*, *TMEM140*, *TRAFD1*, *TRIM14*, *TRIM21*, *TRIM25*, *TRIM26*, *TRIM5*, *TXNIP*, *UBA7*, *UBE2L6*, *USP18*, and *WARS1*.

Receptor-ligand interactions were calculated with CellPhoneDB (40) with default parameters on subsampled cells from each cell type to have an identical amount of cells for each subtype.

Unsupervised epitope specificities for TCRs were calculated with GLIPH2 (30) (v.0.0.1) with default parameter CD4CD8 as reference sets where clusters with less than 3 TCRs were removed. Supervised epitope specificity of TCRs was calculated using TCRGP (31) with default parameters. The epitope models for TCRGP were directly downloaded from the project’s GitHub page (https://github.com/emmijokinen/TCRGP; accessed on March 2, 2021). The tested epitopes were “GILGFVFTL_cdr3b” (from influenza A M1 antigen), “GLCTLVAML_cdr3b” (EBV BMLF1 antigen), “IPSINVHHY_cdr3b” (CMV p65 antigen), “NLVPMVATV_cdr3b” (CMV p65 antigen), “RAKFKQLL_cdr3b” (EBV BZLF1 antigen), “RPRGEVRFL_cdr3b” (HSV2 B7 antigen), “TPRVTGGGAM_cdr3b” (CMV p65 antigen), and “YVLDHLIVV_cdr3b” (EBV BRLF1 antigen). A cutoff to be used in the annotation was determined to be 0.90, and any scores above that were annotated as specific to that given epitope.

### Bulk TCRβ-seq.

TCRβ-seq from genomic DNA was conducted as previously described using the ImmunoSEQ assay by Adaptive Biotechnologies (75) on bone marrow samples from newly diagnosed patients treated with dasatinib (*n* = 6), imatinib (*n* = 4), or nilotinib (*n* = 3). Nonproductive clonotypes were removed from the analysis. The TCRGP predictions were made as described above.

### Immune cell functional assays.

For T cell stimulation, cryopreserved PBMCs were stimulated with anti–human CD3–APC (0.5:100; BD, cat. 561810), anti–human CD49d (1:1000; BD, cat. 340976), anti–human CD28 (1:1000; BD, cat. 340975), and incubated overnight (16 hours) at 37°C in the presence of CD107a/b-FITC (1:18/1:18; BD, cat. 555800 and cat. 555804). For NK cell stimulation, PBMCs were stimulated with K562 cells at a PBMC/K562 ratio of 10:1 in the presence of CD107a/b-FITC.

After the stimulation, the surface markers were stained with CD45-V500-C (1:100; BD, cat. 655873), CD3-APC, CD4-PerCP, CD8-PeCy7 (0.8:100; BD, cat. 335822), and CD56-PE, and cells were permeabilized with FIXPERM (BD, cat. 554714) according to the manufacturer’s instructions. Following permeabilization, intracellular cytokines (3:100; TNF-α–FITC, BD, cat. 554512) and IFN-γ–FITC (3:100; BD, cat. 554700) and granzyme B–Alexa Fluor 700 (0.9:100; BD, cat. 560213) were stained at room temperature. Unstimulated PBMCs were used as controls and samples were acquired with a BD FACSVerse. Gating strategies are shown in Supplemental Figure 5.

### Jurkat TCR activation assay.

The Jurkat NFAT luciferase reporter cell line was obtained from Signosis (SL-0032-NP). The Jurkat cells were plated at 20,000 cells/well on a black-walled, 384-well plate in RPMI-1640 (Lonza) with 10% FBS, 2 mM L-glutamine, 100 U/mL penicillin, and 100 mg/mL streptomycin. The cells were stimulated with anti-CD3 solution (0.5 μg/mL mouse anti–human CD3 [BD, 555329] and goat anti–mouse IgG [Thermo Fisher Scientific, 31160] at 1:4 ratio) and additionally with anti-CD28 (BD, 340975; 0.5 μg/mL) or IFN-α (R&D Systems, 11100-1; 500 U/mL) in the indicated combinations or left unstimulated (Figure 3G). The cells were also treated with dasatinib at 10 nM, 30 nM, or 100 nM or DMSO as control, resulting in a total volume of 25 μL/well. NFAT activation was measured after 5-hour incubation by adding 25 μL One-Glo (Promega) to the wells and measuring luminescence using a PHERAstar FS plate reader (BMG Labtech). To measure cell viability after 24-hour incubation, 25 μL of CellTiter-Glo (Promega) was added to the wells and luminescence was measured.

The experiment was conducted in 6 wells in each condition, and the experiment was done twice, resulting in 288 unique measurements (6 conditions [no stimulation, CD3 stimulation, CD3 + CD28 stimulation, IFN-α stimulation, IFN-α + CD3 stimulation, and IFN-α + CD3 + CD28 stimulation], 4 treatments [DMSO, 10 nM dasatinib, 30 nM dasatinib, and 100 nM dasatinib], 6 wells, 2 batches).

### Data and code availability.

The processed and raw single-cell data will be found in the European Genome-Phenome Archive (EGAS00001005049, https://ega-archive.org/studies/EGAS00001005049). Code to reproduce the findings can be found in github.com/janihuuh/cml_007_manu

### Statistics.

Nonparametric tests were used throughout unless otherwise stated, including the Mann-Whitney *U* test in comparisons between 2 groups or Fisher’s exact test where the alternative hypotheses are reported; and Kruskal-Wallis in comparisons between 3 or more groups. Adjustment for multiple testing was performed when the number of tests exceeded 20, and were either done with Benjamini-Hochberg correction or with Bonferroni’s correction in the DEG analysis. Nominal *P* values and adjusted *P* values less than 0.05 were considered significant. All calculations were done with R (4.0.0) or Python (3.7.4) (https://www.python.org/downloads/). In the box-and-whisker plots, the center line corresponds to the median, the box corresponds to the interquartile range (IQR), and whiskers 1.5 × IQR, while outlier points are plotted individually where present.

### Study approval.

All patients and healthy controls gave their written informed consent, and the study was approved by local University Hospitals and conducted in accordance with the Declaration of Helsinki.

## Author contributions

JH, MI, and SM designed the study, coordinated the project, analyzed the data, and wrote the manuscript. BY analyzed the data. OMJD, HL, TK, and JK performed laboratory analysis. HHH and UOS coordinated the clinical study and participated in the study design. JS, JR, PK, MH, SS, A Dreimane, KP, TGD, BTG, FG, LS, KME, BM, AL, A Dimitrijevic, LU, OWB, HHH, and UOS treated the patients, provided study samples, and collected data. The sequence of first authors was based on the overall merit on the final manuscript. All authors read and approved the final manuscript.

## Supplementary Material

Supplemental data

Supplemental table 1

Supplemental table 2

Supplemental table 3

Supplemental table 4

Supplemental table 5

Supplemental table 6

Supplemental table 7

## Version 1 09/01/2022

Electronic publication
