# Supplementary material for: IFN-α with dasatinib broadens the immune repertoire in patients with chronic-phase chronic myeloid leukemia
Source: J Clin Invest. 2022 Sep 1;132(17):e152585. doi: 10.1172/JCI152585 (PMC9433106; doi:10.1172/JCI152585)
Supplement: Supplemental data [file jci-132-152585-s007.pdf]

S1

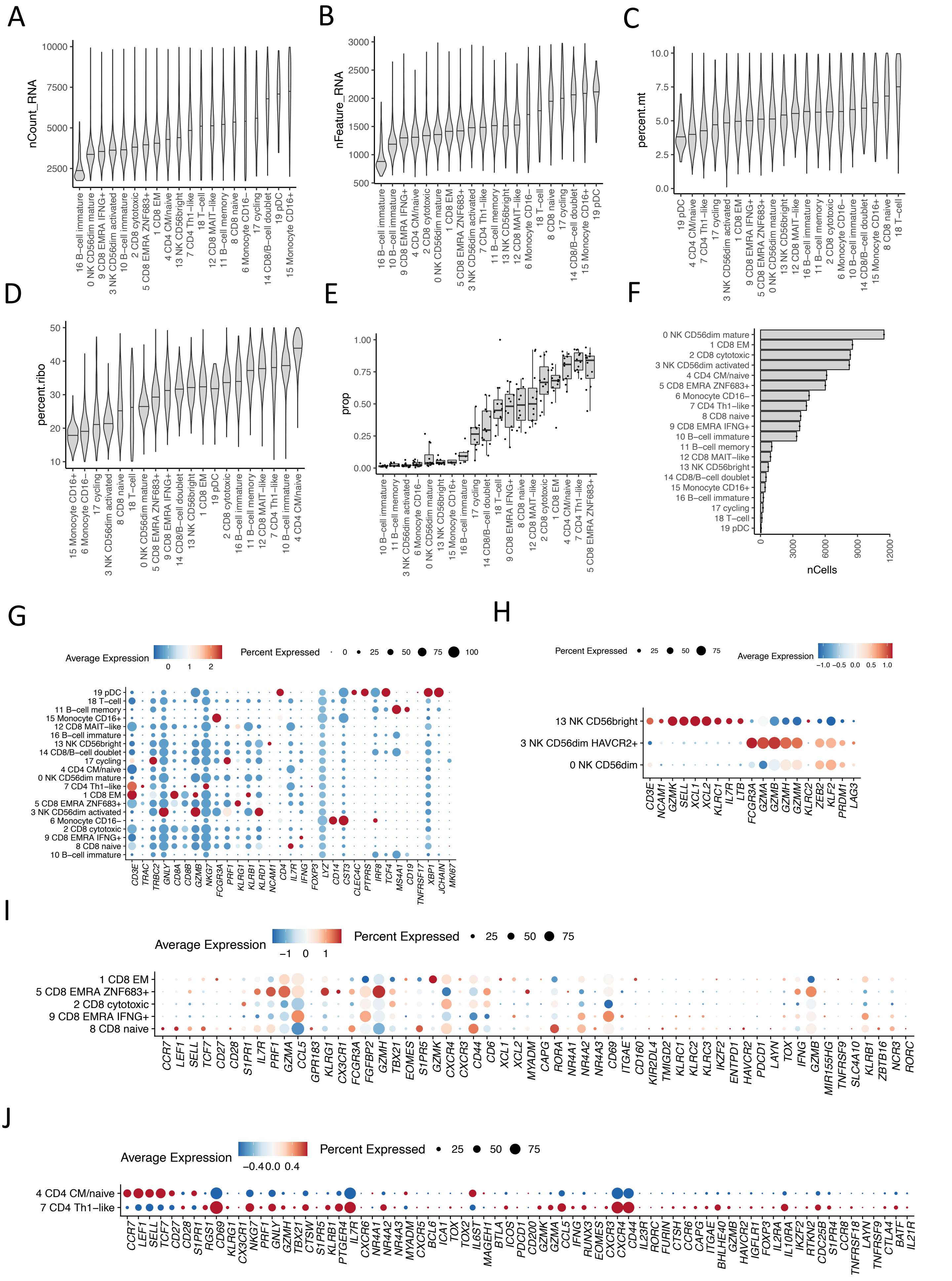

Supplementary Figure 1: Quality control and annotation of scRNAseq clusters
Violin plot showing the **A)** amount of different RNA counts, **B)** the number of genes expressed, **C)** percentage of mitochondrial transcripts, **D)** percentage of ribosomal transcripts. **E)** The abundance of cells with detected TCRα, TCRβ, or TCRαβ. **F)** Bar plot showing the number of cells in each cluster. **G)** Dot plot showing the expression the canonical markers to define clusters broadly (**G**) and to more precisely the NK-cell clusters (**H**) and T-cell clusters (**I** and **J**) . The size reflects the percent of cells in a given cluster expressing the given gene and the color reflects the normalized and scaled average expression of the gene in the cluster.

S2

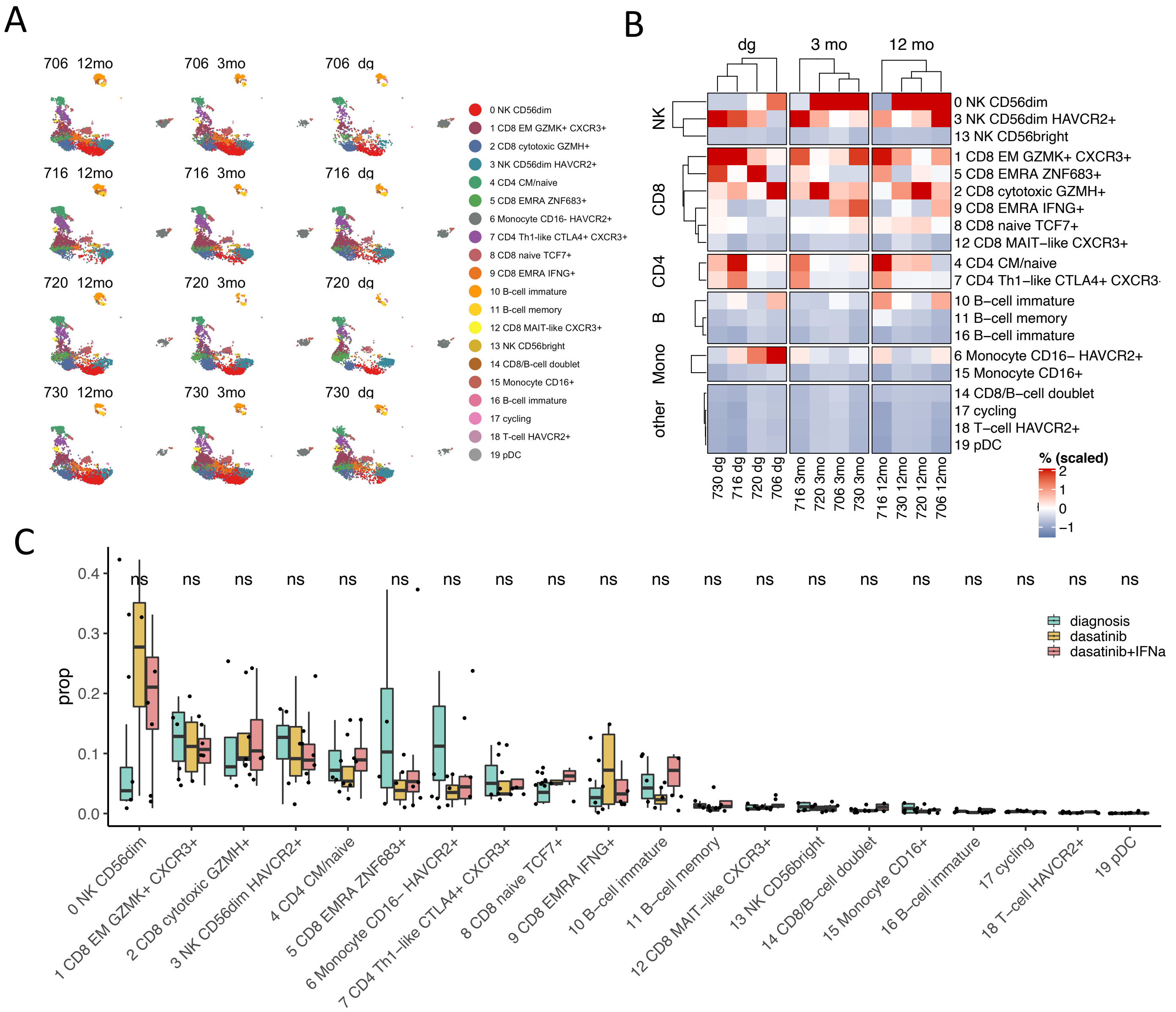

Supplementary Figure 2: The abundances of scRNAseq clusters

A) The same UMAP projection as in **Figure 1A** showing the single-cell RNA sequencing profiles from all 100,00 cells (n=4, 3 time points) in the study, colored by inferred cluster and divided by individual samples. **B)** Heatmap showing the cluster abundancies in individual samples as scaled percentages. The clustering was done with Ward's linkage. **C)** Cluster abundancies shown as box plots in different time points. P-values were calculated with a Kruskal-Wallis test.

S3

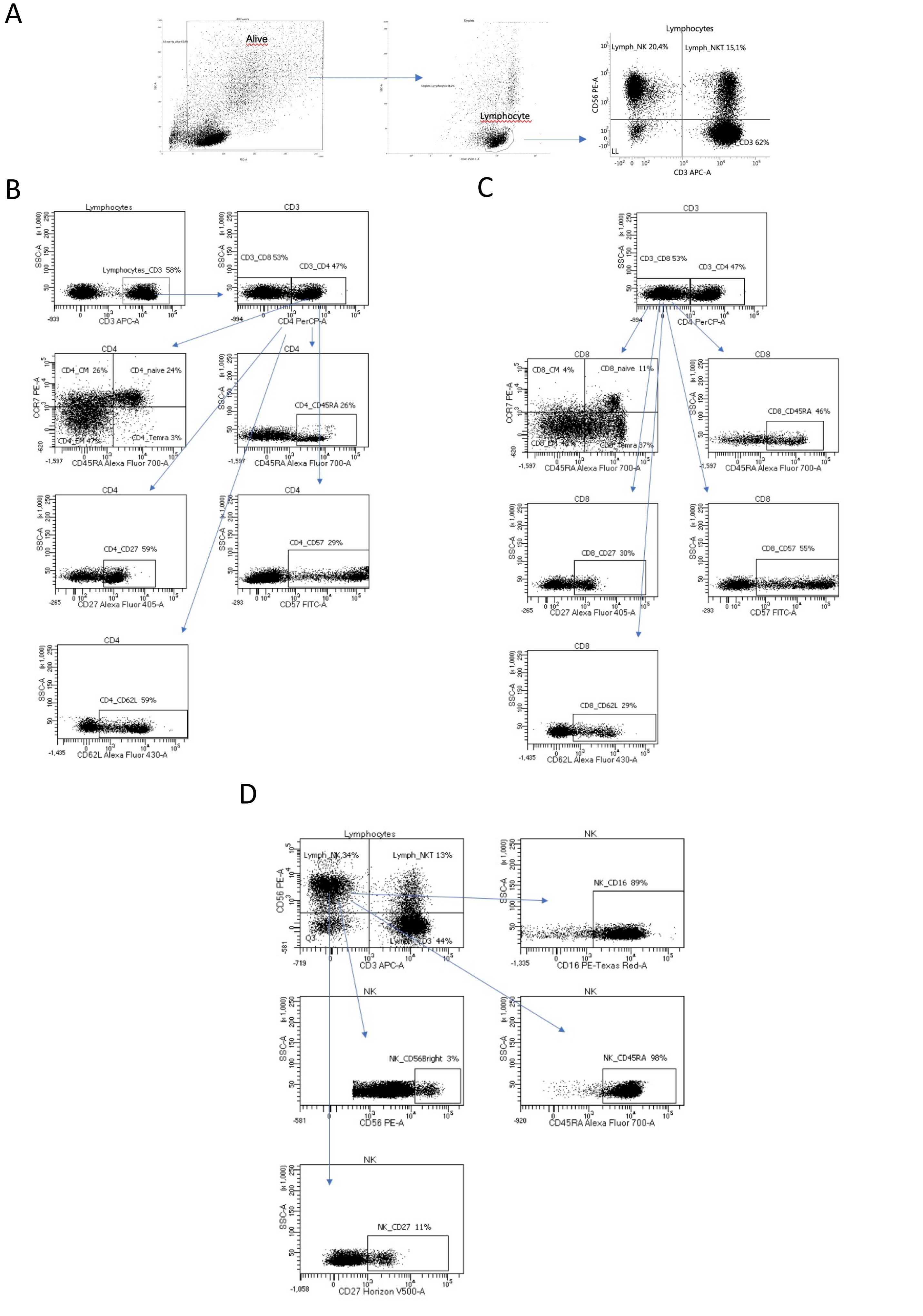

Supplementary Figure 3: Gating strategies for flow cytometry cohorts

Gating strategies for **A)** alive lymphocyte cells, **B)** CD4pos lymphocytes, **C)** CD8pos lymphocytes, and **D)** NK lymphocytes. **A)** First, alive cells are gated from SSC vs FSC scatter, then lymphocytes are gated from SSC vs CD45 scatter. T, NK, and NKT cells are defined based on CD3 and CD56 expressions. **B)** CD3+ T cells are gated from lymphocytes and CD4+ T cells from CD3+ T cells. Naïve CD4+ T cells are defined as CCR7+CD45RA+, central memory (CM) CCR7+CD45RA-, effector memory (T_{EM}) CCR7-CD45RA- and effector memory RA+ (T_{EMRA}) CCR7-CD45RA+. CD27, CD57, and CD62L positive cells are gated from the total CD4+ population. **c)** CD3+ T cells are gated from lymphocytes and CD8+ T cells are defined as CD4 - CD3+ T cells. Naive CD8+ T cells are defined as CCR7+CD45RA+, central memory (CM) CCR7+CD45RA-, effector memory (T_{EM}) CCR7-CD45RA- and effector memory RA+ (T_{EMRA}) CCR7-CD45RA+. CD27, CD57, and CD62L positive cells are gated from the total CD8+ population. **D)** NK cells (CD3-CD56+) are gated from lymphocytes. CD16, CD56bright, CD45RA, and CD27 positive cells are gated from the total NK cell population.

S4

A

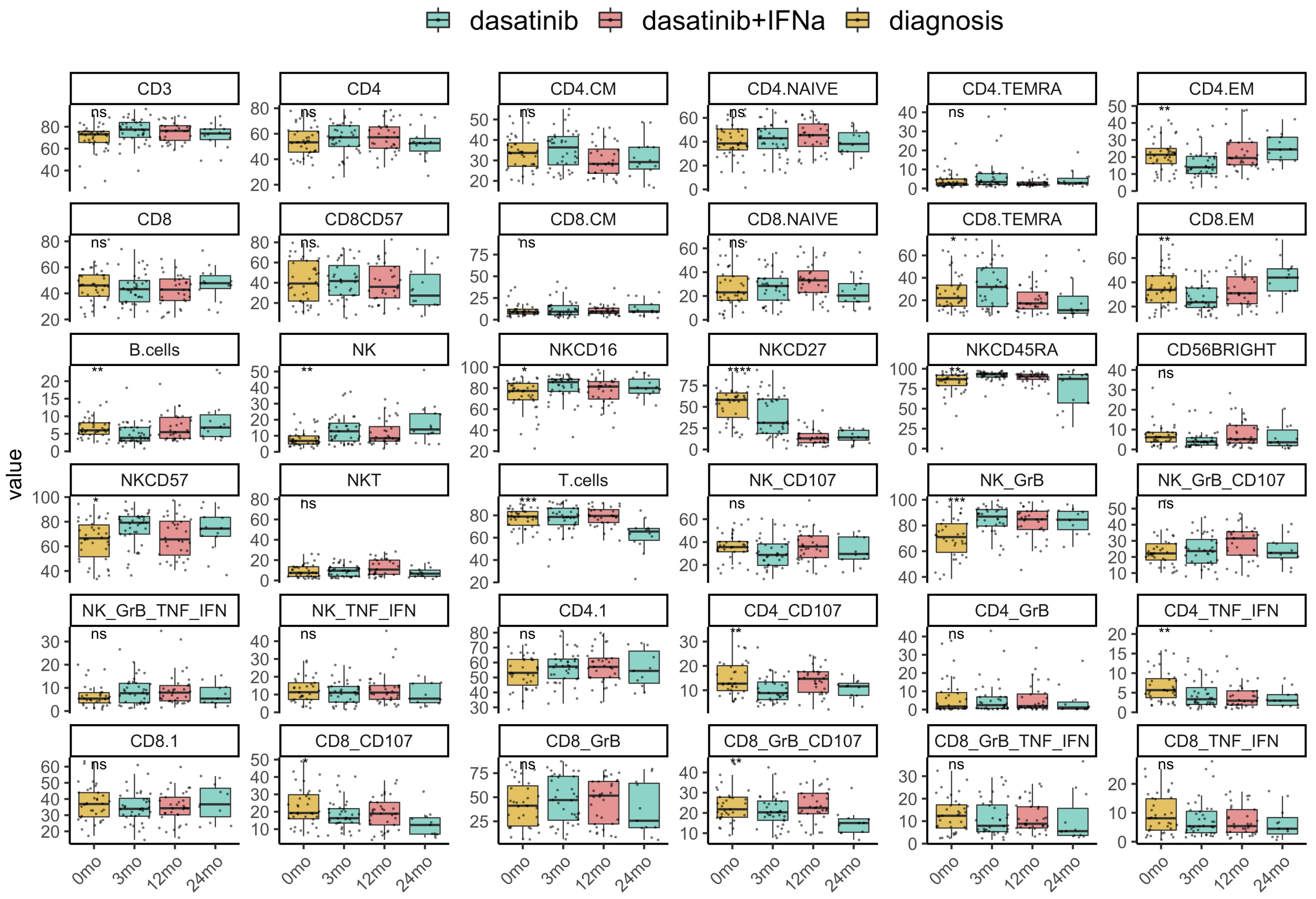

Supplementary Figure 4: The effect of dasatinib, IFN-α, and dasatinib+IFN-α treatments in cell type abundancies

A) The proportion of different immune cell populations profiled with flow cytometry (*n*=40). Populations with two markers (e.g., CD8 CD57) denote the proportion of positive cells from host population (i.e., CD57+ cells from CD8+ cells). Populations with single marker (e.g., T-cells) denote the proportion of these cells from lymphocytes. Populations with “abs” denote absolute cell numbers. More detailed cell populations and *P*-values are shown in **Supplementary Table 3**.

S5

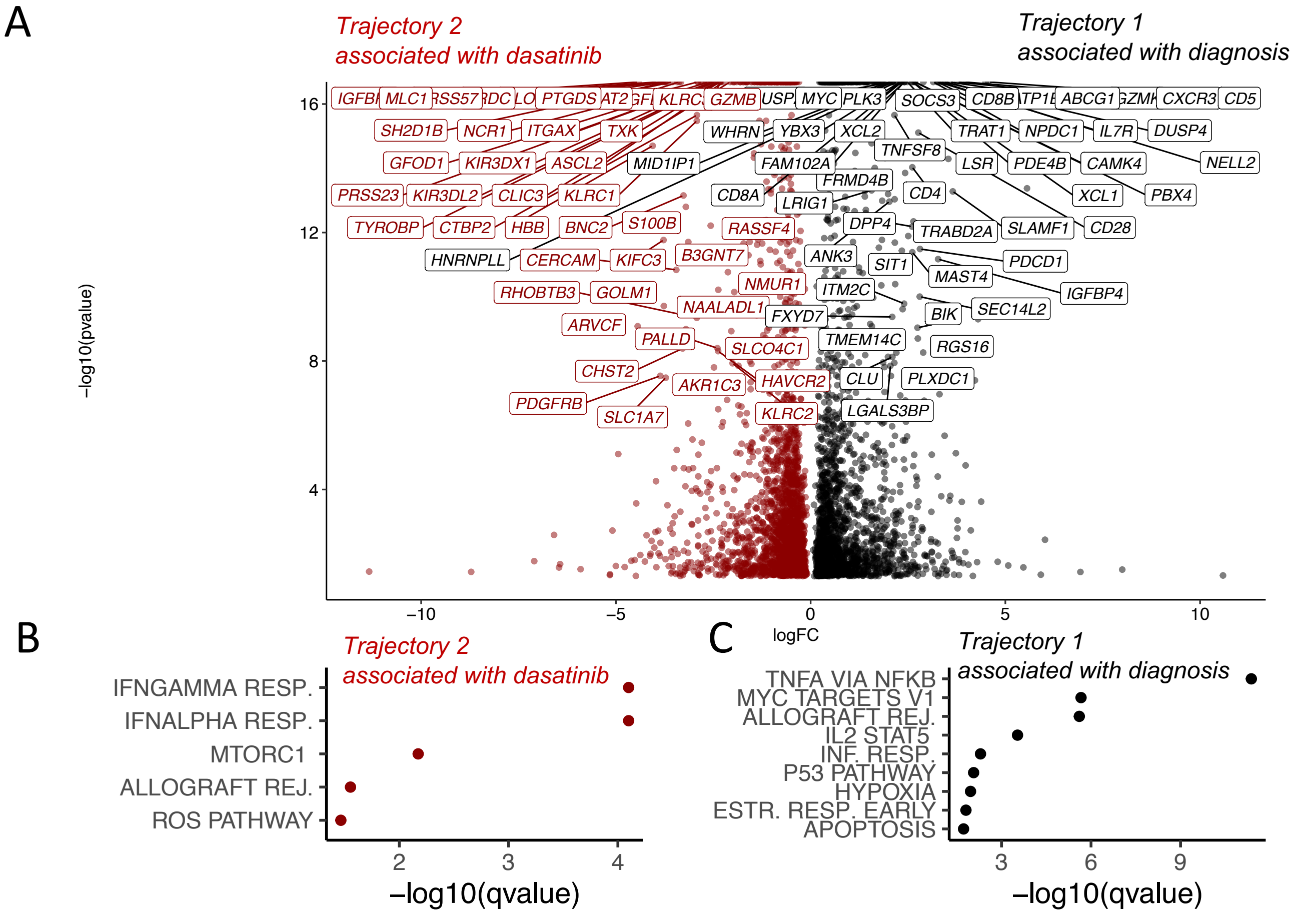

Supplementary Figure 5: Differentially expressed genes between the two trajectories in CD8+ T cells
A) Volcano plot showing the differentially expressed genes between the two CD8+ T cell trajectories presented in **Figure 2H**, calculated with tradeSeq. The labeled genes are the top 50 genes with the smallest *P*-values in each trajectory. Dot plot showing the enrichment of differentially expressed genes to different HALLMARK-categories **B)** trajectory 2, associated with dasatinib, and **C)** trajectory 1, associated with diagnosis. The Q-values are Benjamini-Hochberg corrected Fisher's one-sided exact test *P*-values.

S6

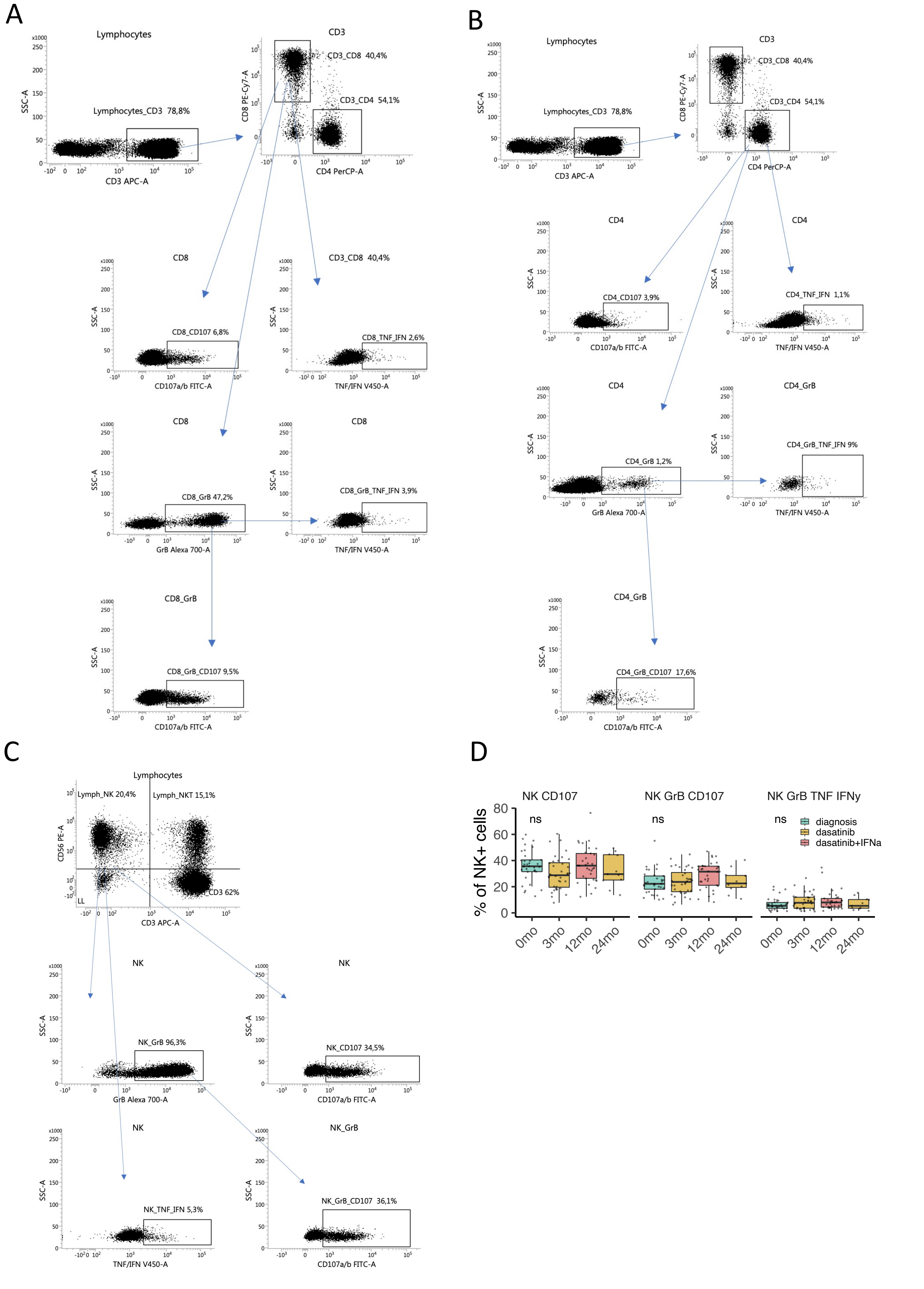

Supplementary Figure 6: Gating strategies for functional analyses

Gating strategies for functional analyses with **A)** CD8+ lymphocytes, **B)** CD4+ lymphocytes, and **C)** NK cells. **A)** CD3+ T cells are gated from lymphocytes and CD8+ T cells from CD3+ T cells. CD107, Granzyme B (GrB), and TNF-α/IFN-γ positive cells are gated from the total CD8+ population. TNF-α/IFN-γ and CD107 positive cells are also gated from the CD8+GrB+ population. **B)** CD3+ T cells are gated from lymphocytes and CD4+ T cells from CD3+ T cells. CD107, GrB, and TNF-α/IFN-γ positive cells are gated from the total CD4+ population. TNF-α/IFN-γ and CD107 positive cells are also gated from the CD4+GrB+ population. **C)** NK cells (CD3-CD56+) are gated from lymphocytes. CD107, GrB, and TNFα/IFNγ positive cells are gated from the total NK cell population. CD107 positive cells are also gated from the GrB+ NK cell population. **D)** Cell type abundances of degranulating (CD107+) and TNF-α releasing NK-cells after being stimulated with CML cell line K562.

S7

A

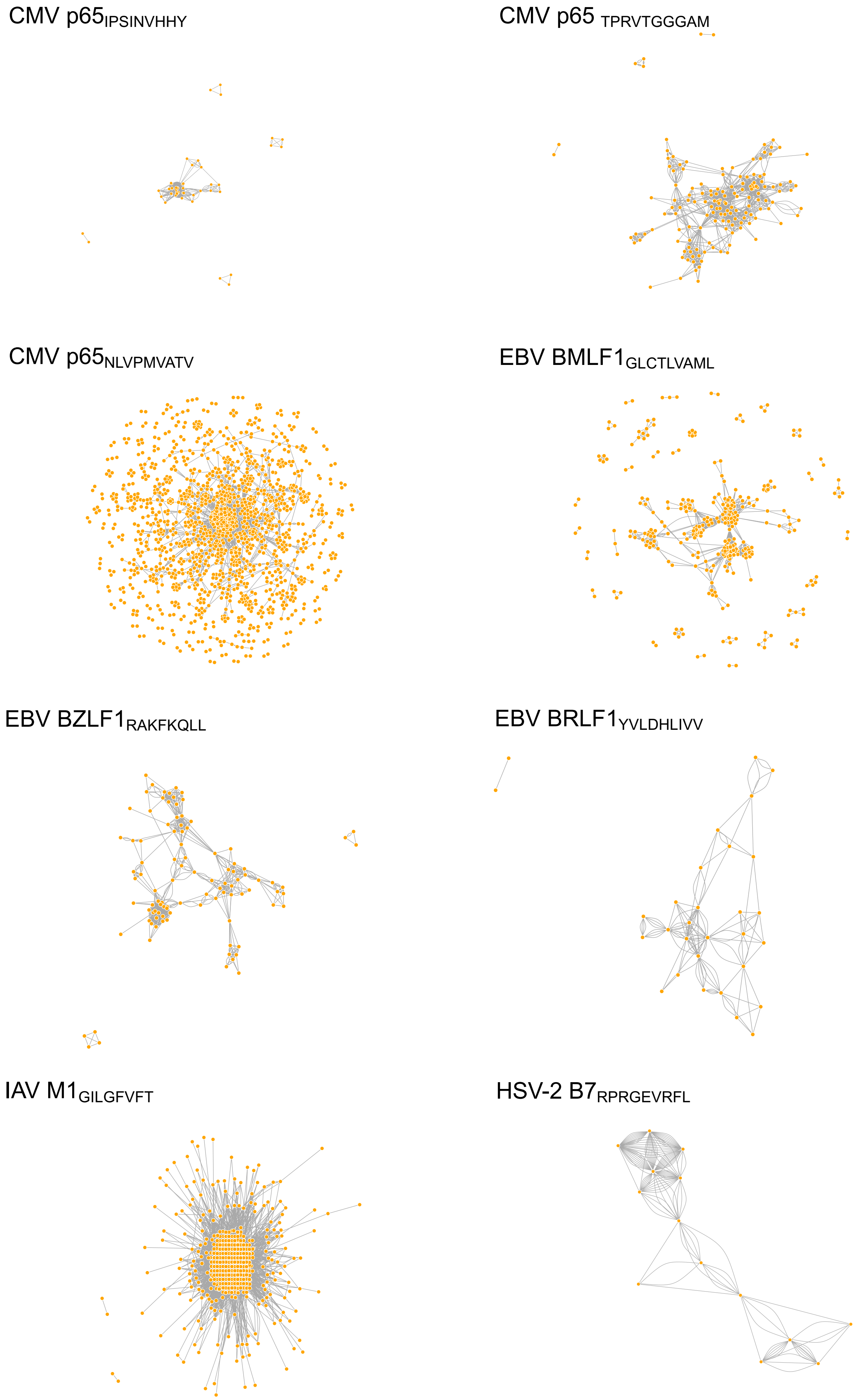

Supplementary Figure 7: Network plots of epitope-specific TCRβ-seq data

A) Network plot showing the epitope-specific groups predicted by GLIPH2. Each node is a T-cell receptor, and the edges denote a GLIPH2 predicted shared epitope-specific target. The different panels correspond to different epitope-specific TCRβ-seq data sets gathered from VDJdb, which were also used as the training data for the TCRGP classifiers.

S8

A

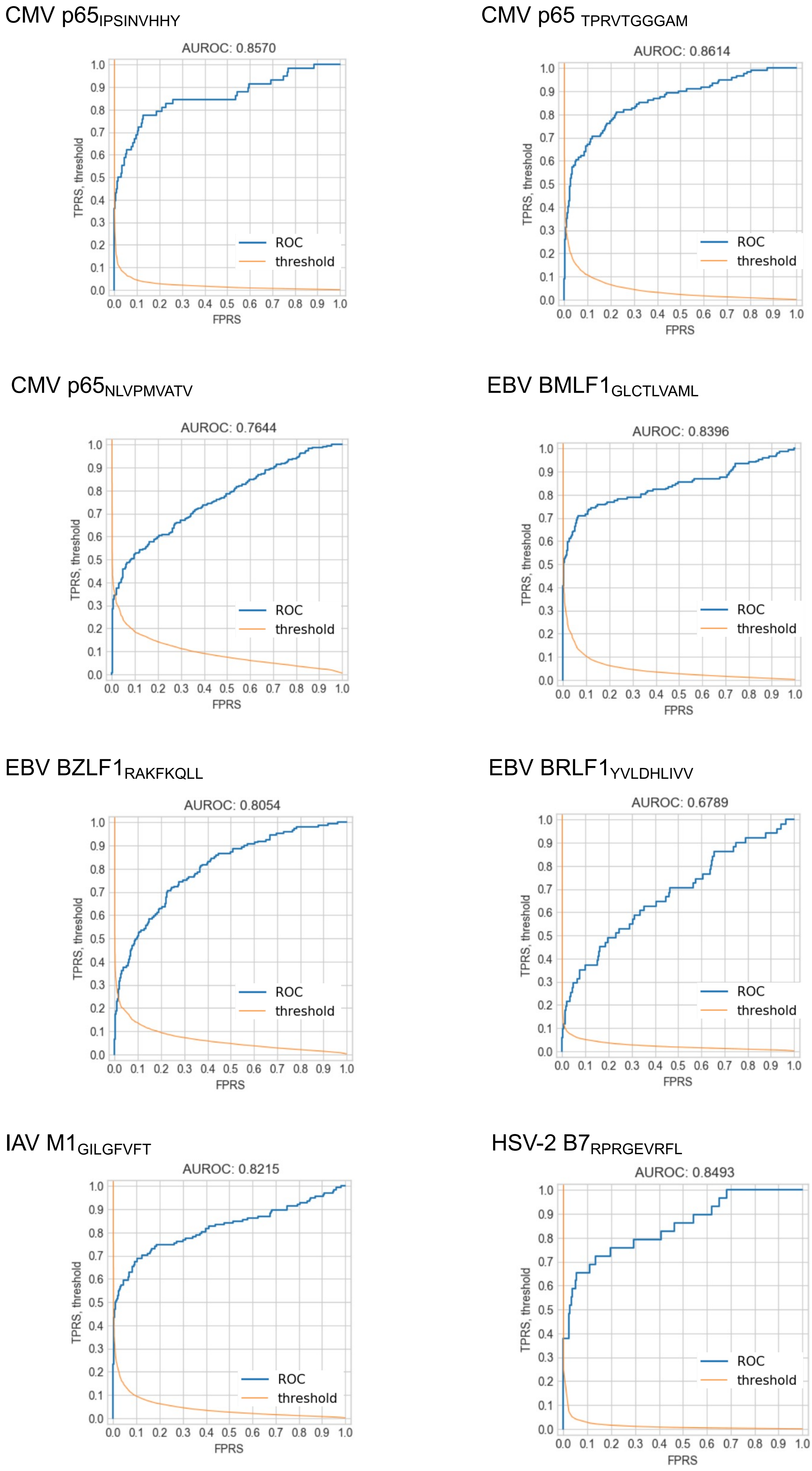

Supplementary Figure 8: Area-under-the-curve (AUROC) figures of TCRGP classifiers on the epitope-specific TCRβ-seq data

A) Area-under-the-curve (AUROC) plots (blue) from different epitope-specific TCRβ-seq data sets gathered from VDJdb for different TCRGP classifiers used in the study. The used threshold (orange) for TCRGP classifiers can be determined from these plots by assessing the different true positive rates (TPRS) and false positive rates (FPRS).

S9

A

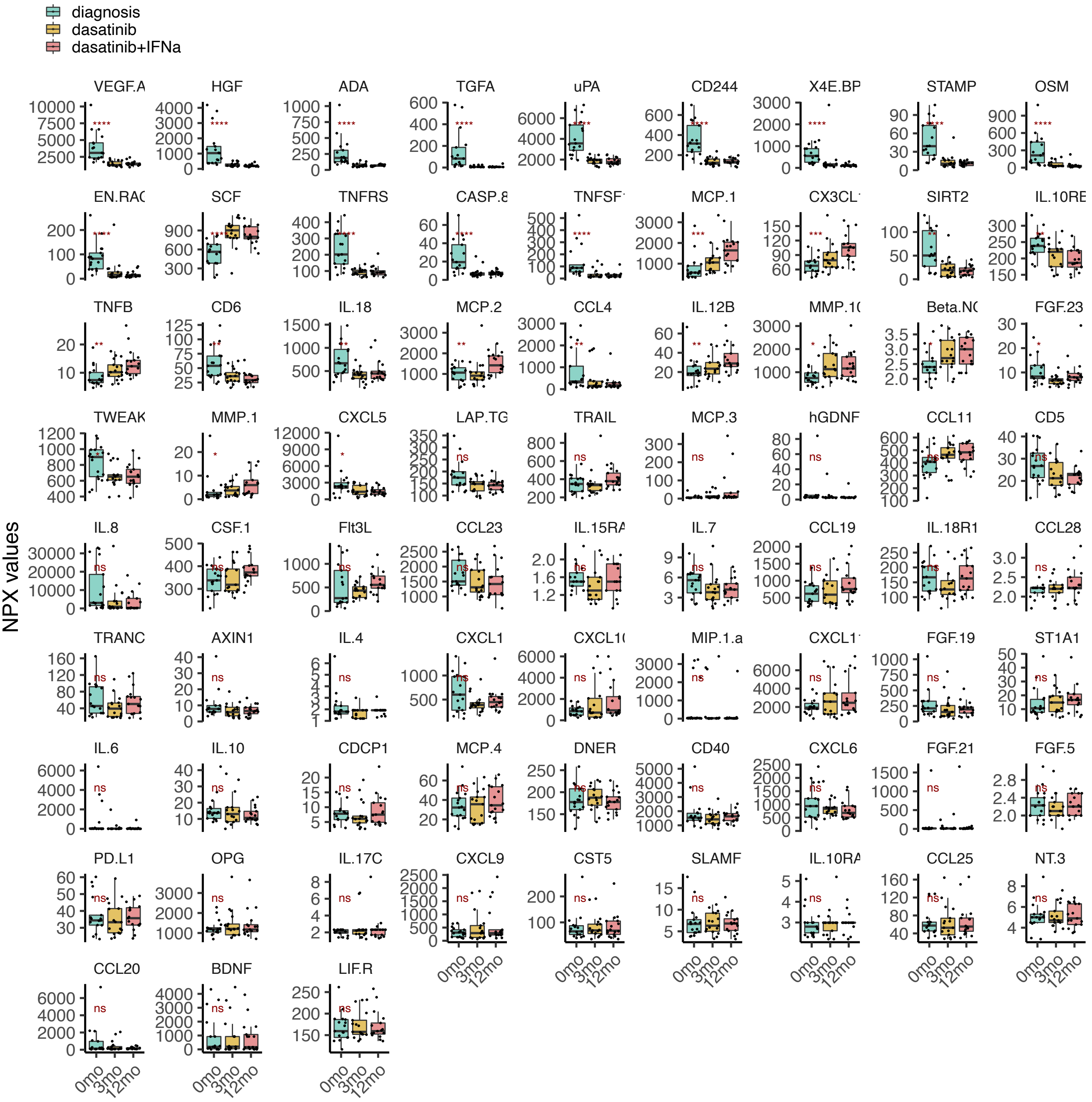

Supplementary Figure 9: The effect of dasatinib, IFN-α, and dasatinib+IFN-α treatments on cytokine levels

A) The expression of different statistically differentially expressed (P_{adj}<0.05, Benjamini-Hochberg corrected Kruskal-Wallis) cytokines. *P*-values were calculated with a Kruskal-Wallis test.

S10

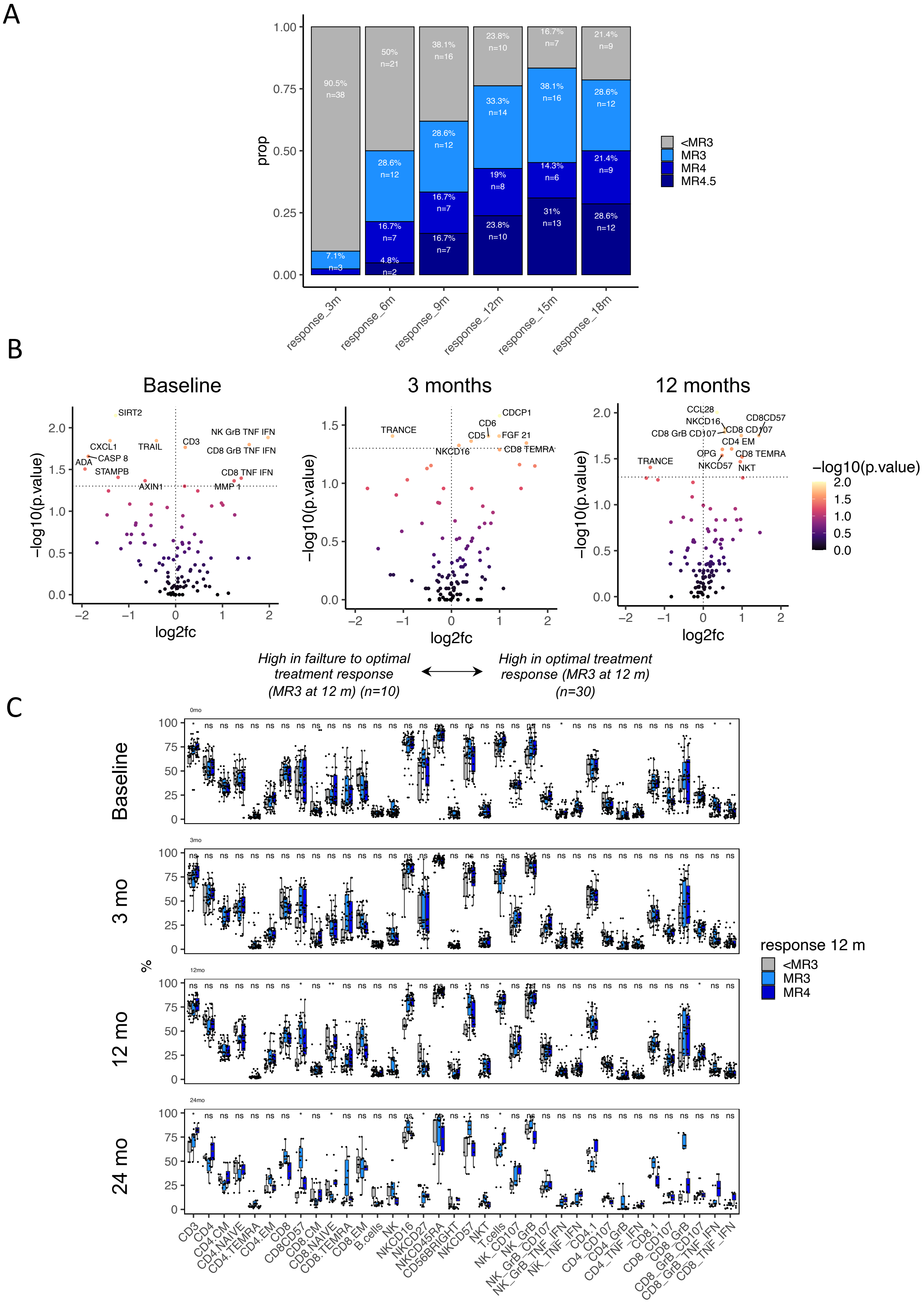

Supplementary Figure 10: Correlations of immunological parameters with treatment responses
A) Bar plot showing the proportion of patients in different clinical responses in different time points. <MR3=*BCR-ABL1*^{IS}>0.1%, MR3=*BCR-ABL1*^{IS}<0.1%, MR4=*BCR-ABL1*^{IS}<0.01%, and MR4.5=*BCR-ABL1*^{IS}<0.0032%. MR4 category includes patients with MR3 and MR4, and MR4.5 category includes patients with MR3, MR4, and MR4.5. **B)** Volcano plot showing the differentially abundant (*P*<0.05, Mann-Whitney test) immune cell populations in the flow cytometry cohort and the differentially expressed cytokines between the patients with early treatment success (defined as <0.1% *BCR-ABL1*^{IS} levels at 12 months) to patients failing to reach this milestone. The immunologic parameters (flow cytometry population abundances and cytokines) have been measured at different time points. The X-axis denotes the log2 fold-change of median population abundance in clusters, where higher values denote upregulation in patients with optimal treatment response. **C)** The proportion of different immune cell populations profiled with flow cytometry (*n*=40) divided by the response status at 12 months. Populations with two markers (e.g., CD8 CD57) denote the proportion of positive cells from host population (i.e., CD57+ cells from CD8+ cells). Populations with single marker (e.g., T-cells) denote the proportion of these cells from lymphocytes. Populations with “abs” denote absolute cell numbers. More detailed cell populations are shown in **Supplementary Table 3**. *=*P*<0.05, **= *P*<0.01, ***= *P*<0.001, ****= *P*<0.0001

S11

a

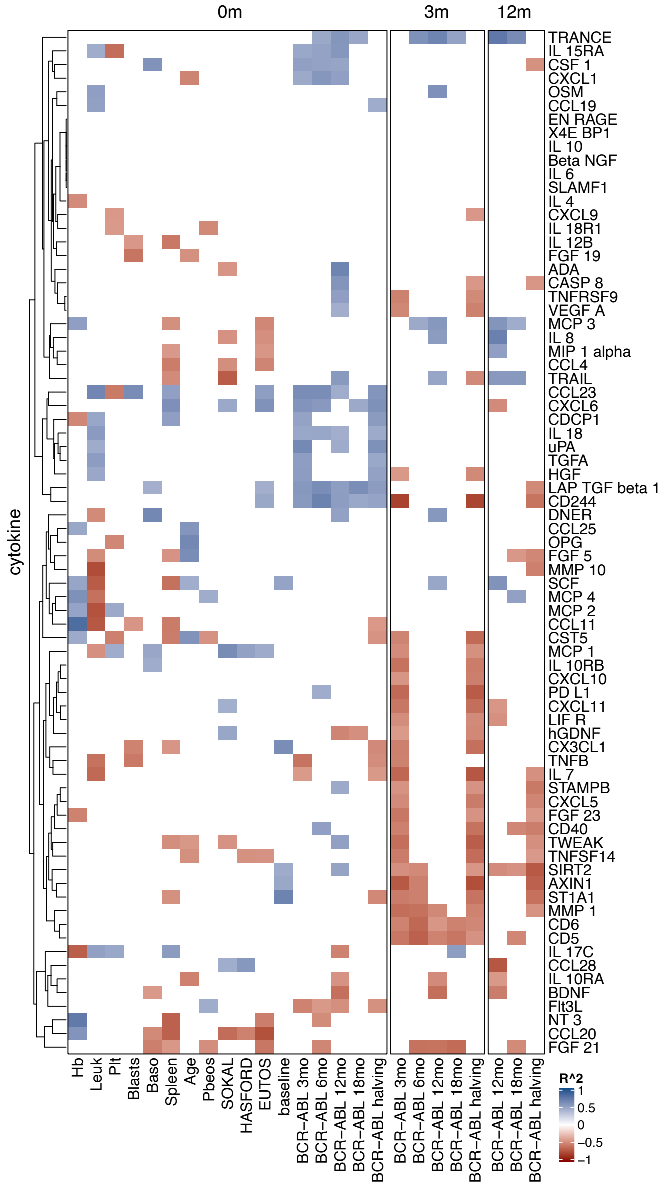

Supplementary Figure 11: Clinical correlations with cytokines

A) Heatmap showing the R^2 values from Spearman's rank correlation analysis of cytokine expression values (NPX) with the clinical parameters. Only correlations with P<0.1 are shown.

Supplementary Table legends:

Supplementary Table 1: The characteristics of flow cytometry, scRNA+TCRαβ-, and TCRβ-sequenced patients

Supplementary Table 2: Differentially expressed genes and pathways of the single-cell RNA sequencing data at various time points

Supplementary Table 3: *P*-values for the flow cytometry and cytokine profile data at various time points

Supplementary Table 4: Unsupervised TCRαβ targets predicted by GLIPH2

Supplementary Table 5: Supervised TCRαβ targets predicted by TCRGP

Supplementary Table 6: Significant immune interactions predicted by CellPhoneDB

Supplementary Table 7: *P*-values for the flow cytometry and cytokine profile correlated with clinical covariates
